# Diversity and Temporal Dynamics of the Epiphytic Bacterial Communities Associated with the Canopy-Forming Seaweed *Cystoseira compressa* (Esper) Gerloff and Nizamuddin

**DOI:** 10.3389/fmicb.2016.00476

**Published:** 2016-04-08

**Authors:** Francesco P. Mancuso, Sofie D'Hondt, Anne Willems, Laura Airoldi, Olivier De Clerck

**Affiliations:** ^1^Dipartimento di Scienze Biologiche, Geologiche ed Ambientali, Centro Interdipartimentale di Ricerca per le Scienze Ambientali, UO CoNISMa, University of BolognaRavenna, Italy; ^2^Phycology Research Group and Center for Molecular Phylogenetics and Evolution, Ghent UniversityGhent, Belgium; ^3^Laboratory for Microbiology, Department of Biochemistry and Microbiology, Ghent UniversityGhent, Belgium

**Keywords:** epiphytic bacteria communities, high throughput sequencing, 16S rRNA gene, canopy-forming seaweeds, Fucales, *Cystoseira compressa*, Mediterranean Sea

## Abstract

Canopy-forming seaweed species of the genus *Cystoseira* form diverse and productive habitats along temperate rocky coasts of the Mediterranean Sea. Despite numerous studies on the rich macrofauna and flora associated with *Cystoseira* spp., there is little knowledge about the epiphytic bacteria. We analyzed bacterial populations associated with canopies of *Cystoseira compressa*, over an annual vegetative cycle (May-October), and their relationships with the bacterial populations in the surrounding seawater, at intertidal rocky shores in Vasto (Chieti—Italy). The bacterial diversity was assessed using Illumina Miseq sequences of V1-V3 hypervariable regions of 16S rRNA gene. *C. compressa* bacterial community was dominated by sequences of *Proteobacteria* and *Bacteroidetes, Verrucomicrobia, Actinobacteria*, and *Cyanobacteria* especially of the *Rhodobacteriaceae, Flavobacteriaceae, Sapropiraceae, Verrucomicrobiaceae*, and *Phyllobacteriaceae* families. Seawater libraries were also dominated by *Proteobacteria* and *Bacteroidetes* sequences, especially of the *Candidatus Pelagibacter* (SAR11) and *Rhodobacteriaceae* families, but were shown to be clearly distinct from *C. compressa* libraries with only few species in common between the two habitats. We observed a clear successional pattern in the epiphytic bacteria of *C. compressa* over time. These variations were characterized by gradual addition of OTUs (*Verrucomicrobia, Actinobacteria* and SR1) to the community over a growing season, indicative of a temporal gradient, rather than a radical reorganization of the bacterial community. Moreover, we also found an increase in abundance over time of *Rhodobacteraceae*, comprising six potential pathogenic genera, *Ruegeria, Nautella, Aquimarina, Loktanella, Saprospira*, and *Phaeobacter* which seemed to be associated to aged thalli of *C. compressa*. These bacteria could have the potential to affect the health and ecology of the algae, suggesting the hypothesis of a possible, but still unexplored, role of the microbial communities in contributing to the extensive ongoing declines of populations of *Cystoseira* spp. in the Mediterranean Sea.

## Introduction

Canopy seaweeds of the genus *Cystoseira* C. Agardh (Fucales, Phaeophyceae) are among the most important habitat-forming species in the Mediterranean Sea. With the majority of its species endemic to the Mediterranean Sea (Ribera et al., 1992; Gómez-Garreta et al., 2002; Draisma et al., 2010), *Cystoseira*-dominated vegetations provide food and protection for rich associated communities, comprising other algae, invertebrates and fish (Mineur et al., 2015). In addition *Cystoseira* stands significantly enhance the structural complexity and productivity of coastal communities from the infralittoral down to the upper circalittoral zone (Giaccone et al., 1994; Bulleri et al., 2002; Falace and Bressan, 2006; Ballesteros et al., 2009).

During the last decades several *Cystoseira* species have retracted their ranges considerably to the point where several species have been reported to be locally lost (Soltan et al., 2001; Thibaut et al., 2005, 2015; Serio et al., 2006; Mangialajo et al., 2007, 2008; Perkol-Finkel and Airoldi, 2010). The loss of *Cystoseira* canopies leads to structurally less complex communities most often dominated by low-lying, turf-forming species (Benedetti-Cecchi et al., 2001; Connell et al., 2014) or sea urchin barrens (Agnetta et al., 2015). These shifts are attributable to the interactive effects of different local and global stressors (Asnaghi et al., 2013; Strain et al., 2014, 2015).

The ecological responses of seaweeds to most abiotic and biotic stressors are perceived and transmitted through the algal surface, which represents a highly active interface between the seaweed and the environment. The surface is involved in exchange processes such as the uptake and release of nutrients, waste products and secondary metabolites. Bacteria, which typically form biofilms on the algal surface, are hypothesized to affect the interactions between the seaweeds and the environment by modifying the properties of the external surfaces (Wahl et al., 2012). Bacteria interact with seaweeds, thereby modulating the health, performance and resilience of their hosts. Biofilms can reduce the access of their hosts to light, gases and nutrients and alter the interaction with other fouling epibionts, consumers and pathogens (Goecke et al., 2010; Wahl et al., 2012). The tight relationship between seaweeds and microbiota renders these associations functionally equivalent to a single entity, or a holobiont (Egan et al., 2013). Although a growing number of papers focus on the bacterial communities associated with different seaweeds (e.g., Bengtsson et al., 2012; Wahl et al., 2012; Hollants et al., 2013; Miranda et al., 2013; Campbell et al., 2015), the underlying mechanisms of these associations remain largely unknown.

Recent investigations present a major stride toward documenting the phylogenetic composition of associated bacterial communities and their spatio-temporal dynamics (e.g., Staufenberger et al., 2008; Bengtsson et al., 2010; Burke et al., 2011). Most studies concur that algal-associated bacterial communities are distinct from the surrounding environment and largely host-specific (Lachnit et al., 2009). Nevertheless, bacterial communities display considerable temporal and spatial variation (Campbell et al., 2015; Fuhrman et al., 2015). There is growing evidence that the communities of surface bacteria are highly influenced by the physiology of the host. Bengtsson et al. (2010) demonstrated that assembly and dynamics of the biofilm is correlated with the growth cycle of *Laminaria.* More recently, observations that microbial communities were more strongly associated with host condition (healthy versus stressed) in the brown alga *Ecklonia radiata* than with geographical location or environmental variables, highlights that host traits may be a critical determinant of the associated microbial community structure (Marzinelli et al., 2015). Despite these reports, the functional relationships with the host species remain largely an open question. Understanding the dynamics of epiphytic bacteria would allow to explore potentially overlooked mechanisms behind algal responses to environmental or anthropogenic stressors.

We characterized the composition and dynamics of epibiotic bacteria of the canopy-forming seaweed *Cystoseira compressa* (Esper) Gerloff and Nizamuddin along an intertidal rocky promontory in the southern Adriatic Sea. We used next generation sequencing Illumina Miseq of 16S rRNA gene libraries to characterize the diversity (richness, evenness, and community composition) of bacterial communities and describe their successional changes over a vegetative growth season. We also tested whether bacterial communities associated to *C. compressa* were distinct from those found in the surrounding seawater, to explore potential specificities toward the *C. compressa* host.

## Materials and methods

### Study area and species

**Figure 1:**
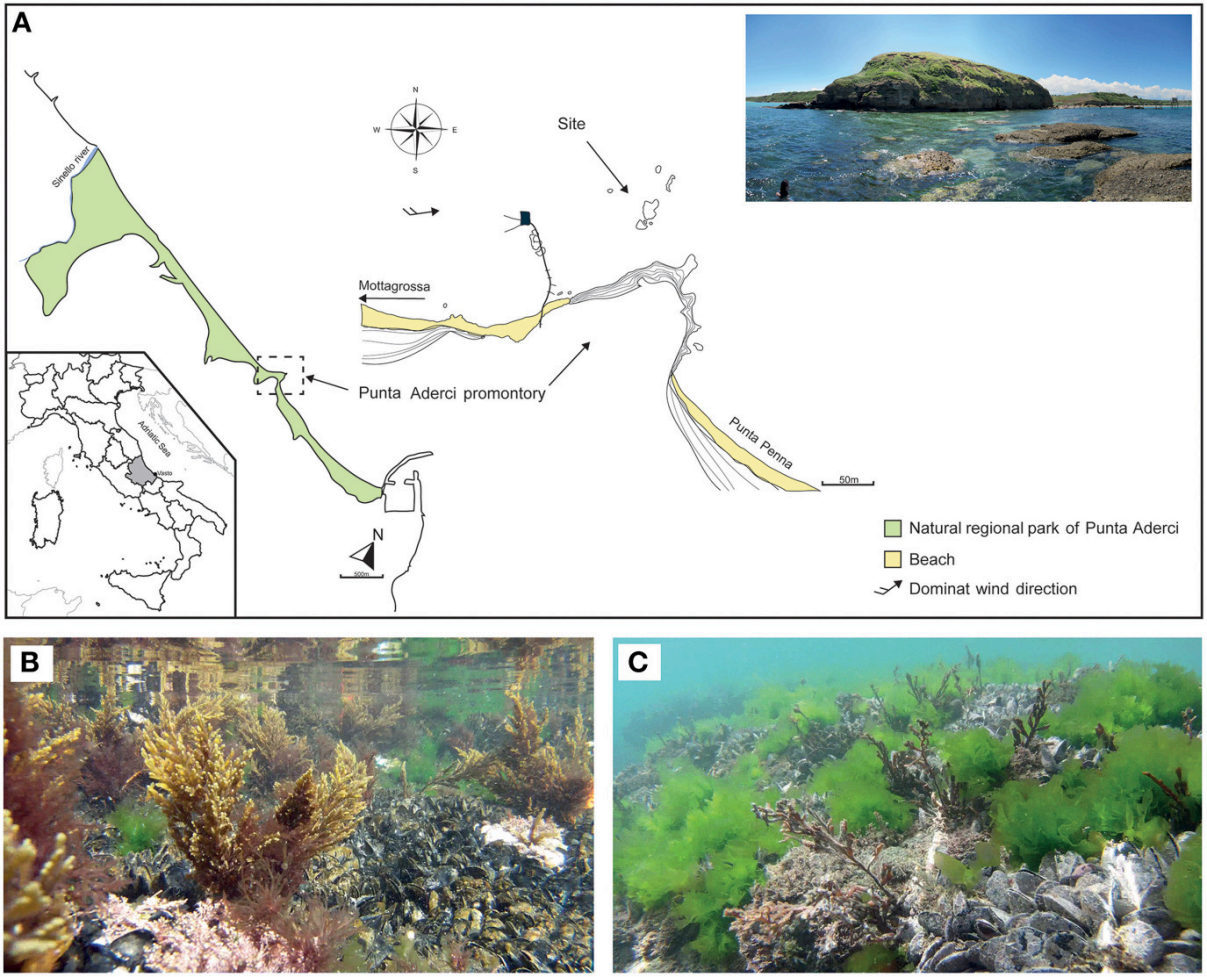
**Main physiographic characteristics of the coastal area and sampling site of the Punta Aderci promontory (A)**. Underwater assemblage characterized by *Mytilus galloprovincialis, Ulva rigida* and *Cystoseira compressa* during summer **(B)** and late summer **(C)**.

*Cystoseira compressa* and associated microbial communities were sampled along the rocky shore at Punta Aderci promontory, Vasto, Italy (42°10′50.3″ N, 14°41′15.0″ E) in the central Adriatic Sea (Figure 1A). This promontory, situated in the central sector of Abruzzo coast, is characterized by clay–sand–conglomerate lithotypes (Miccadei et al., 2011), moderate exposure to wave action and an average tidal amplitude of ≈ 30 cm. We sampled populations of *C. compressa* at the sublitoral fringe (−10 cm to + 10 cm relative to Mean Low Water Level; MLWL). Seawater temperature ranges from a minimum of 8°C in winter to 27.5°C in summer (data from the “Istituto Superiore per la Protezione e Ricerca Ambientale,” ISPRA, period 2000–2013, www.mareografico.it). The underwater rocky substrate is dominated by patches of mussels (*Mytilus galloprovincialis)*, ephemeral algae (*Ulva rigida)*, and perennial stands of *C. compressa* (Figures 1B,C). *C. compressa* is the only canopy-forming alga in this habitat. Like other species of *Cystoseira, C. compressa* exhibit pronounced seasonal variations in vegetative growth (Gómez-Garreta et al., 2002; Falace et al., 2005). At the study location, new branches develop from a perennial basis in May, providing a fresh substrate for colonizing bacteria. In July, thalli reach their maximum height and physiological activity (Figure 1B) while in late August *C. compressa* loses most upright branches (Figure 1C). The basal cauloid persists in a quiescent state during the cold winter season.

### Sampling

Bacterial communities were collected from submerged thalli of *C. compressa* and the surrounding seawater six times from May to October 2014 during the vegetative growth season (Table S1). Each time, epiphytic bacteria were collected from 3 randomly selected thalli of *C. compressa*. Sterile cotton swabs on wooden sticks (Aptaca) were used to rub approximately 12 cm^2^ of surface from the perennial base to the tip of primary branches. Swabs were immediately placed in sterile 1.5 ml Eppendorf tubes. Thalli that were overgrown with epiphytic seaweeds or animals were avoided. To compare the bacterial communities that grow on *C. compressa* with those present in the surrounding environment, two samples (*n* = 2) of seawater (500 ml) were randomly collected each time using black polyethylene bottles (Kartell). Seawater samples were filtered in the field with an electric vacuum pump, connected to a portable electric generator, first onto 3.0 μm pore size cellulose acetate filters (Millipore), to remove most eukaryotes, and then onto 0.2 μm pore size cellulose nitrate filters (Sartorius) to retain the bacteria. Samples were transported on ice to the lab and stored at −80°C until DNA extraction.

### Characterization of the bacterial community

Microbial DNA was extracted from the swabs using a protocol from Zwart et al. (1998). Briefly, the tip of the swab was placed into a 2 ml tube with 0.5 g of zirconium beads (0.1 mm diameter) to lyse the cells, 0.5 ml 1X TE buffer (10 mM Tris, pH 8) and 0.5 ml buffered phenol (pH 7–8) were added to the tubes containing the swab tips and the tubes were vigorously shaken (30 Hz) on a Bead-beater (Retsch) three times for 2 min with intermittent cooling on ice. The tubes were then centrifuged for 5 min at 10,000 rpm in a cooled centrifuge (4°C) and the upper (aqueous) phase was transferred to a new tube and extracted with phenol-chloroform- isoamylalcohol (25:24:1). The DNA was then precipitated by adding 1/10 volume of 3 M sodium acetate (pH 5) and 2 volumes of 96% (v/v) ethanol and incubating overnight at −20°C. Subsequently, the DNA was washed with ethanol 70% (v/v) and dissolved in 1xTE buffer. The V1-V3 region of the bacterial 16S rRNA gene was amplified using forward pA (AGAGTTTGATCCTGGCTCAG 8–27) (Edwards et al., 1989) and reverse BKL1 1 (GTATTACCGCGGCTGCTGGCA 536– 516; Cleenwerck et al., 2007) primers. PCR reaction mixes were made using the Faststart High Fidelity PCR system (Roche). The PCR mix consisted of: nuclease-free water; reaction buffer 1x; 0.8 mM of each dNTP; 0.5 μM of each primer; 0.02 U *Taq* (FastStart Taq DNA Polymerase); approximately 30 ng template DNA. PCR conditions were: 94°C for 5 min, 30 cycles of 94°C for 30 s, 50°C for 30 s, 72°C for 30 s, and final elongation at 72°C for 7 min. Libraries for Illumina MiSeq v3 (2 × 300 bp) were constructed using the NexteraXT DNA sample preparation kit with a dual indexing strategy consisting of two 8-base indices. Amplicons obtained from the first PCR were cleaned using Ampure beads. Then we performed a second PCR, with 12 cycles, to attach the adaptors and the indexes on the amplicons obtained previously. After a further clean up with Ampure beads and equimolar pooling the samples were sent for sequencing.

The microbial amplicon sequences were processed using the UPARSE pipeline (Edgar, 2013), implemented in the USEARCH package version 8.0.1623 (Edgar, 2010), unless stated otherwise. Paired-end reads with a minimum length overlap of 60 bp were merged, discarding reads with a length shorter or longer than 450 and 530, respectively. Moreover, no gaps were allowed in the alignment of the overlapping region. The reads were quality-filtered by imposing a maximum expected error of 0.5. Samples were pooled and truncated using the *trim.seqs* function in Mothur (Schloss et al., 2009). After dereplication, singletons were discarded, and sequences were binned into OTUs with a minimum identity of 97%. Chimeric sequences were detected with the UCHIME algorithm (Edgar et al., 2011) using the RDP gold database (training database v9) as a reference. Taxonomy assignment was performed in QIIME 1.9.0 using the Greengenes 16S rRNA gene dataset (13_8_99 release; DeSantis et al., 2006) with RDP classifier method (Wang et al., 2007) and a confidence value of the 0.8. The sequences were classified from phylum to genus level. After classification chloroplast and mitochondrial sequences were removed from the dataset. Moreover, samples with a library size smaller than 1000 sequences were removed, because samples below this level can suffer from quality issues (Navas-Molina et al., 2013). Finally, to correct for possible contamination during the lab work, OTUs detected in the negative control were removed from the data set. For phylogenetic tree reconstruction, sequences were aligned with Clustal Ω with default parameters for nucleotide alignment. The phylogenetic tree was reconstructed using the gamma model of sequence evolution (options “-nt -gamma -no2nd -fastest -spr 4”) in FastTree2 (Price et al., 2010; Hildebrand et al., 2014). Statistical analyses were performed in R software 3.1.2 (R Core Team, 2015) using the “*phyloseq*” R-package (McMurdie and Holmes, 2013).

Microbial composition was described from phylum to genus level. First, the relative abundance of each OTU within each sample was calculated, then the OTUs were sorted in descending order according to their relative abundance, and the most abundant ones, comprising at least 90% of the community, were retained. A phylogenetic tree was built and used to show differences between the bacterial communities of *C. compressa* and surrounding seawater. Moreover, the log2 fold change times based on the OTUs abundance data was calculated to show which OTU contributed more in the differences between habitats.

The original output files of each sample have been submitted to the NCBI sequence read archive under the accession SRX1563424. Sequences of all 3820 OTUs (97% clustering) have been submitted to GenBank under the accession numbers KU688205–KU692024.

### Analysis of spatial and temporal variations

We characterized the alpha and beta diversity of the bacterial communities collected from two habitats, *C. compressa* and surrounding seawater, over the six sampling times. To estimate alpha diversity, data sets were rarefied at the number of sequences of the sample with the least sequencing depth. Data were rarefied using the “*rarefy_even_depth*” function in the “*phyloseq*” library (we defined a random number seed to 33, R environment). OTU richness and the Chao1 index were calculated using the “*estimated_diversity*” index in the “*phyloseq*” library, while Shannon-Wiener index was estimated using the “*diversity*” function in the “*vegan*” R-package (Oksanen et al., 2015). Pielou's evenness was calculated as H/ln(S), where S and H are the estimated OTU richness and Shannon-Wiener diversity, respectively. For each habitat and each sampling time, we calculated mean values and standards errors for each of these metrics. Differences in alpha diversity parameters between habitats (2 levels, fixed factor) and sampling times (6 levels, random factor, orthogonal to habitat) were statistically tested by performing univariate permutational analyses of variance (PERMANOVA) with PERMANOVA+ (Anderson et al., 2008) for PRIMER v.6 (Clarke and Gorley, 2006). The analysis was based on a Euclidian distance matrix with type III of sum of squares, 9999 permutations, and unrestricted permutation of raw data. PERMANOVA was chosen for univariate analyses because it allows for two-factor designs, considers an interaction term and does not assume a normal distribution of errors.

Spatial and temporal variations of the bacterial communities structure were displayed by unconstrained ordination plots using the principal coordinate analysis (PCoA), based on a Bray-Curtis distance matrix calculated from the square-root transformed OTU abundance data. Differences between habitats and sampling times of the bacterial communities were statistically tested by using a multivariate PERMANOVA. The PERMANOVA analysis was based on a Bray-Curtis similarity matrix with type III of sum of squares, 9999 permutations and unrestricted permutation of raw data. SIMPER analysis was performed in PRIMER v.6 (Clarke and Gorley, 2006) to identify those OTUs that most characterized the epiphytic bacteria community composition of *C. compressa* at each time or that mostly contributed to the differences observed. Cut-off value was restricted to 60%. To explore how different OTUs contributed to the diversity patterns, bubble plots of the abundances of the main correlated OTUs were plotted on the PCoA graph.

## Results

**Figure 2:**
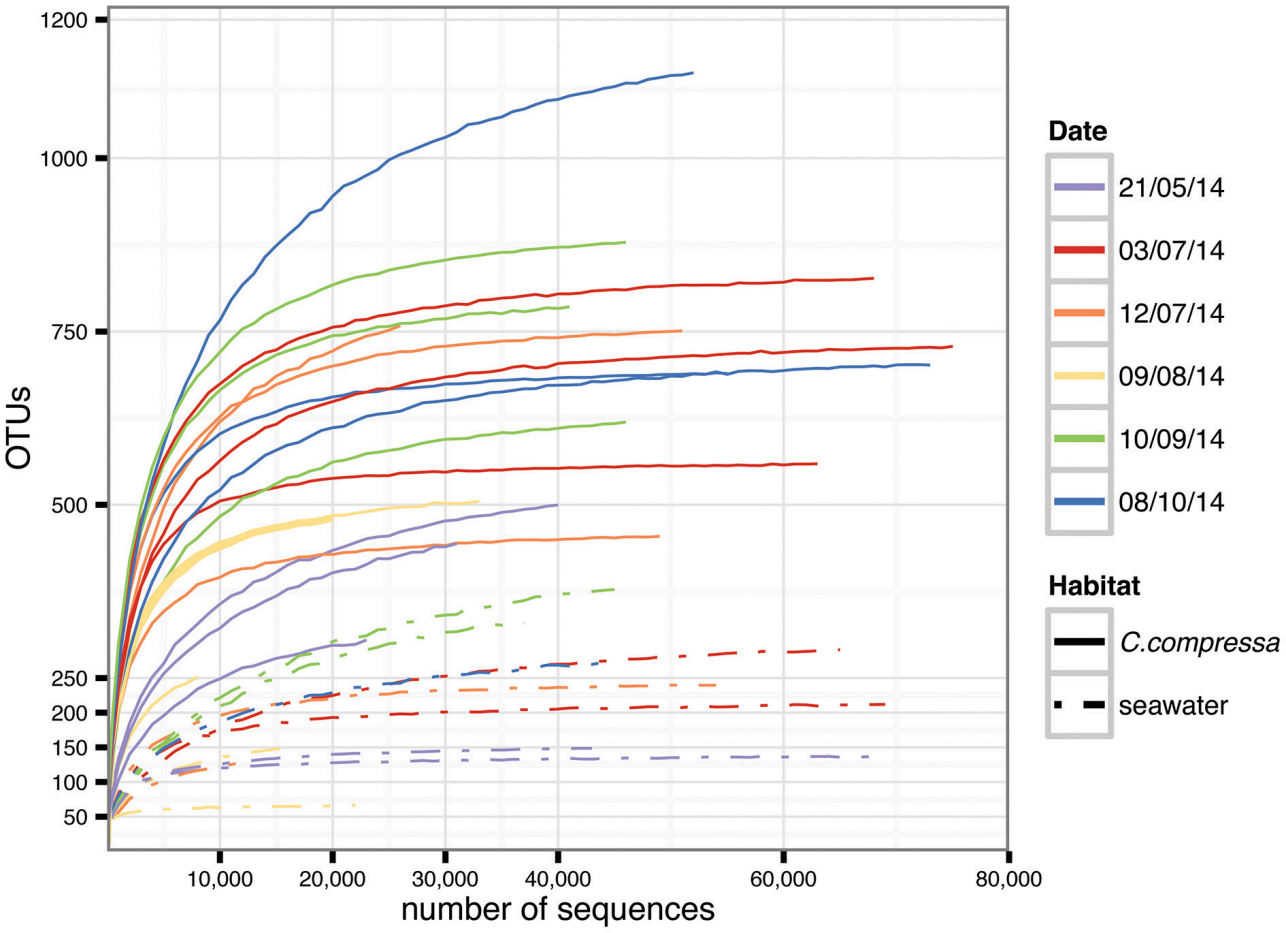
**Rarefaction curves generated for each sample**. Solid and dot-dash lines refer to *Cystoseira compressa* and seawater samples, respectively. Colors represent different time points.

Targeting the hypervariable V1-V3 region of the 16S rRNA, a total of 15,799,968 paired-end raw reads were obtained using the Illumina Miseq v.3 platform. After quality filtering and discarding singletons, chimeras and chloroplast and mitochondrial sequences, our dataset contained 1,289,599 sequences with an average length of 483 ± 5 bp. The average number of reads was 44,469 per library (*SD* = 18,999; min = 8,727; max = 75,903) while the total OTU richness was 3820 at the 97% OTU definition (see Tables S1, S2 for more details). Classification of OTUs against the Greengenes database resulted in 56.9 and 16.2% of the OTUs being classified at family and genus levels, respectively. Classification success increased from 71.9 to 100% with higher taxonomic levels (Figure S1). Rarefaction curves showed saturation for most of the samples, indicative of a good coverage of diversity (Figure 2).

### Bacterial diversity of *C. compressa* and surrounding seawater

**Figure 3:**
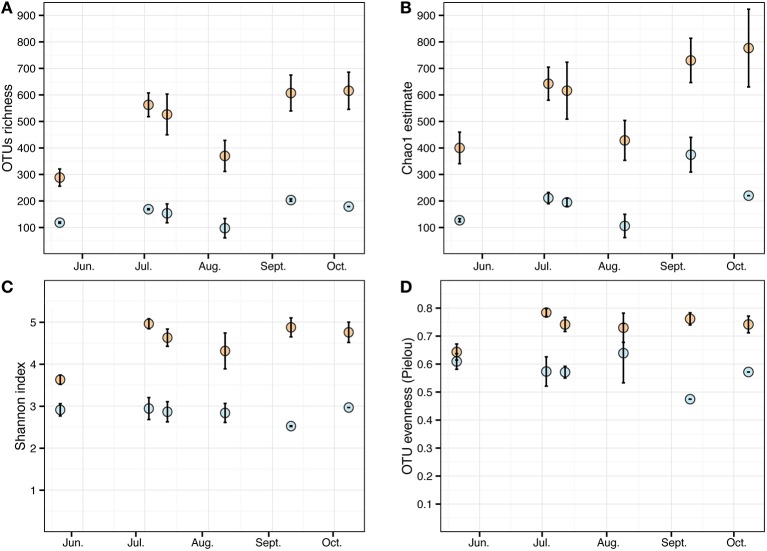
**Alpha diversity measures of the microbial communities associated to *C. compressa* (orange) and surrounding seawater (blue) across seasons**. Observed OTUs **(A)**, Chao1 species richness estimates **(B)**, Shannon diversity H' **(C)**, and Pielou's evenness index **(D)**. Values are means ± standard error (*n* = 3-2).

*Cystoseira*-associated bacterial diversity was significantly higher compared to the surrounding seawater at all sampling times (Figures 3A,B, Table S3). Likewise, the Shannon index was always higher in bacterial communities associated to *Cystoseira* than in the surrounding seawater (Figure 3C). Bacterial evenness was generally high both on *Cystoseira* and in the surrounding seawater, with Pielou's evenness index slightly higher on *Cystoseira* samples (Figure 3D).

**Figure 4:**
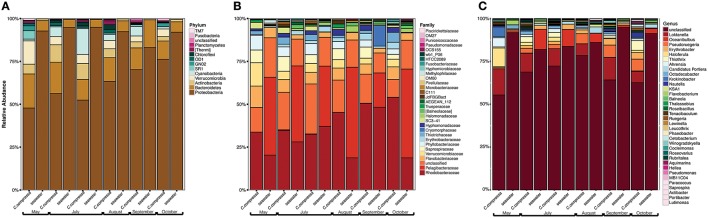
**Pattern of the bacterial communities of *C. compressa* and surrounding seawater across seasons**. Data reported are the relative abundance of the top 300 OTUs accounting for the 92% of the data set at phylum **(A)**, family **(B)**, and genus **(C)** levels.

**Figure 5:**
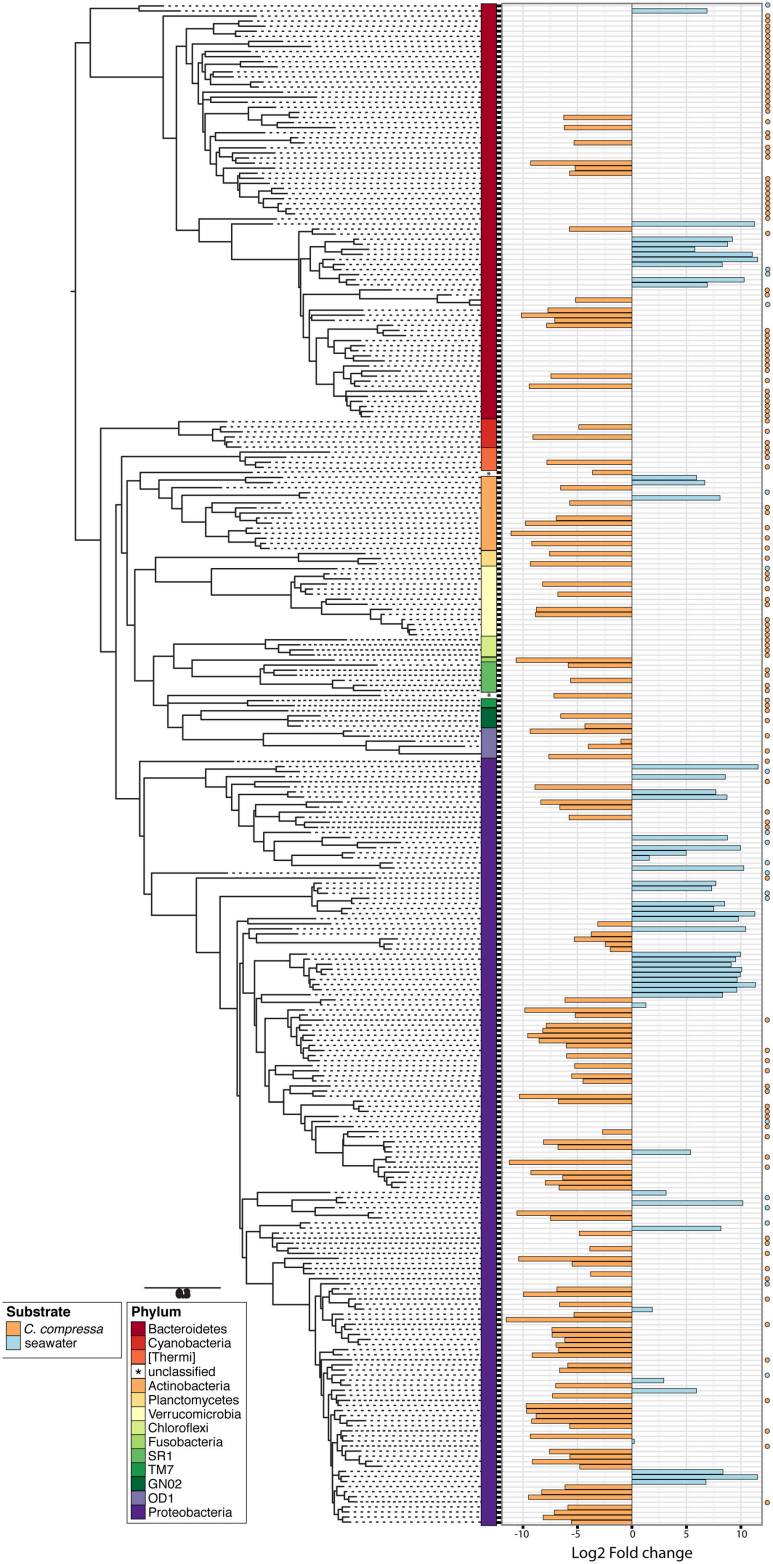
**Phylogenetic tree of the dominant OTUs (*n* = 300) in bacterial communities associated with *C. compressa* and the surrounding seawater**. The color strips denote phylum-level classification. Bar plot shows the Log2 fold change times based on the OTUs abundance on *C. compressa* (orange) and in seawater (blue). Dot points show exclusive species for each habitat.

Phylogenetic characterization identified 33 phyla of Bacteria: 13 of these (*Proteobacteria, Bacteroidetes, Verrucomicrobia, Actinobacteria*, SR1, OD1*, Thermi*, GN02, *Chloroflexi, Planctomycetes*, TM7, *Fusobacteria, Cyanobacteria*), together with the OTUs that could not be classified at phylum level, comprised more than 90% of the diversity in the dataset. *Proteobacteria, Bacteroidetes, Actinobacteria, Verrucomicrobia*, and *Cyanobacteria* were by far the most abundant taxa, accounting for 69.7, 9.7, 2.9, 2.7, and 2.2% of the diversity, respectively (Figure 4A and Table S4). At the family level, most sequences of the epiphytic bacteria on *C. compressa* were classified as *Rhodobacteraceae* (34.7%), *Flavobacteriaceae* (6.6%), *Saprospiraceae*, and *Verrucomicrobiaceae* (5.2% each), while the seawater samples mainly comprised representatives of *Pelagibacteraceae* (40.2%) and *Rhodobacteraceae* (27.6%). About 15–16% of the OTUs, however, remained unclassified at family level in both habitats (Figure 4B and Table S5). At genus level only the 28.6% of the OTUs were classified. *C. compressa* harbored *Loktanella* (8.8%)*, Pseudoruegeria* (3.6%) (family *Rhodobacteraceae*), and *Haloferula* (2.6%) (family *Verrucomicrobiaceae*), while seawater samples showed an important presence of *Oceanibulbus* (5.1%) (family *Rhodobacteraceae*) and *Erythrobacter* (1.2%) (family *Erythrobacteraceae*). However, the high percentage of unclassified OTUs at genus level does not allow providing detailed information of the two habitats at that level (Figure 4C). Of the 13 phyla mentioned above, *Chloroflexi*, and TM7 were exclusively found associated to *C. compressa*. Overall, *C. compressa* hosted a much greater number of exclusive OTUs (121) than seawater (19) (Figure 5).

### Successional changes in epibacterial diversity on *C. compressa* and surrounding seawater

**Figure 6:**
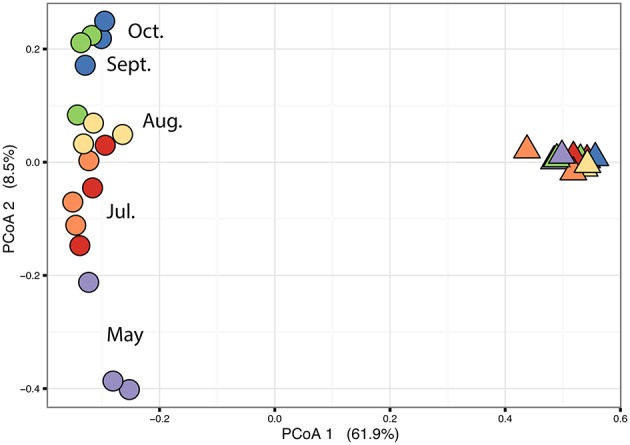
**Principal coordinate analysis plot (PCoA) based on a Bray-Curtis distance matrix calculated from the square-root transformed OTU abundance data of the bacterial community of *C. compressa* and surrounding seawater across times**. Violet, red, orange, yellow, green, and blue points represent the following sample times respectively: 21-05-14, 03-07-14, 12-07-14, 09-08-14, 10-09-14, and 08-10-14.

**Figure 7:**
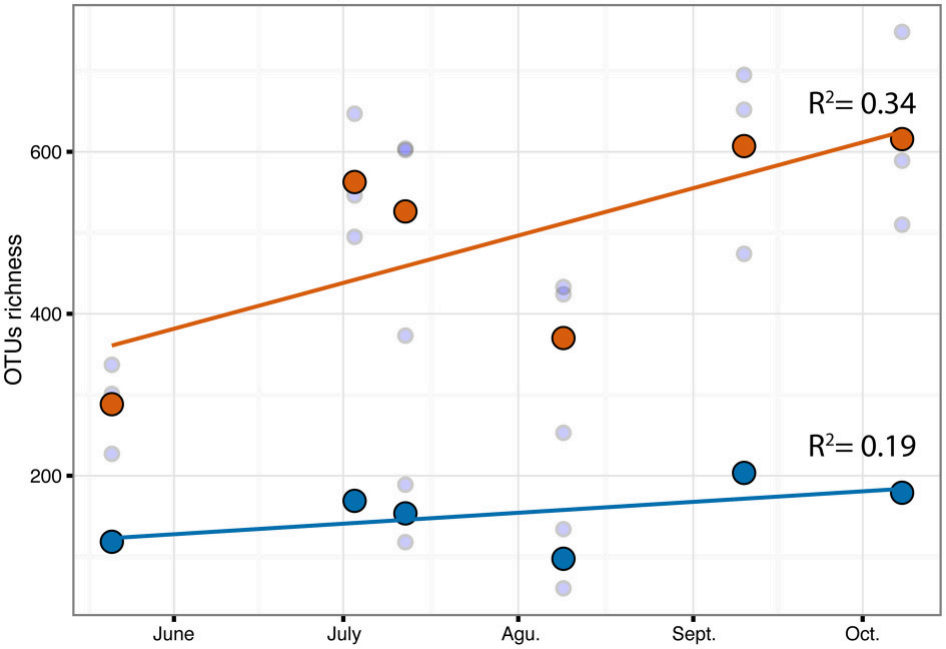
**Correlation of OTUs richness over time for *C.compressa* (orange dots) and seawater (blue dots)**. Dots and lines are mean values and tendency respectively. Values of Pearson correlation (R^2^) are shown inside the plot.

**Figure 8:**
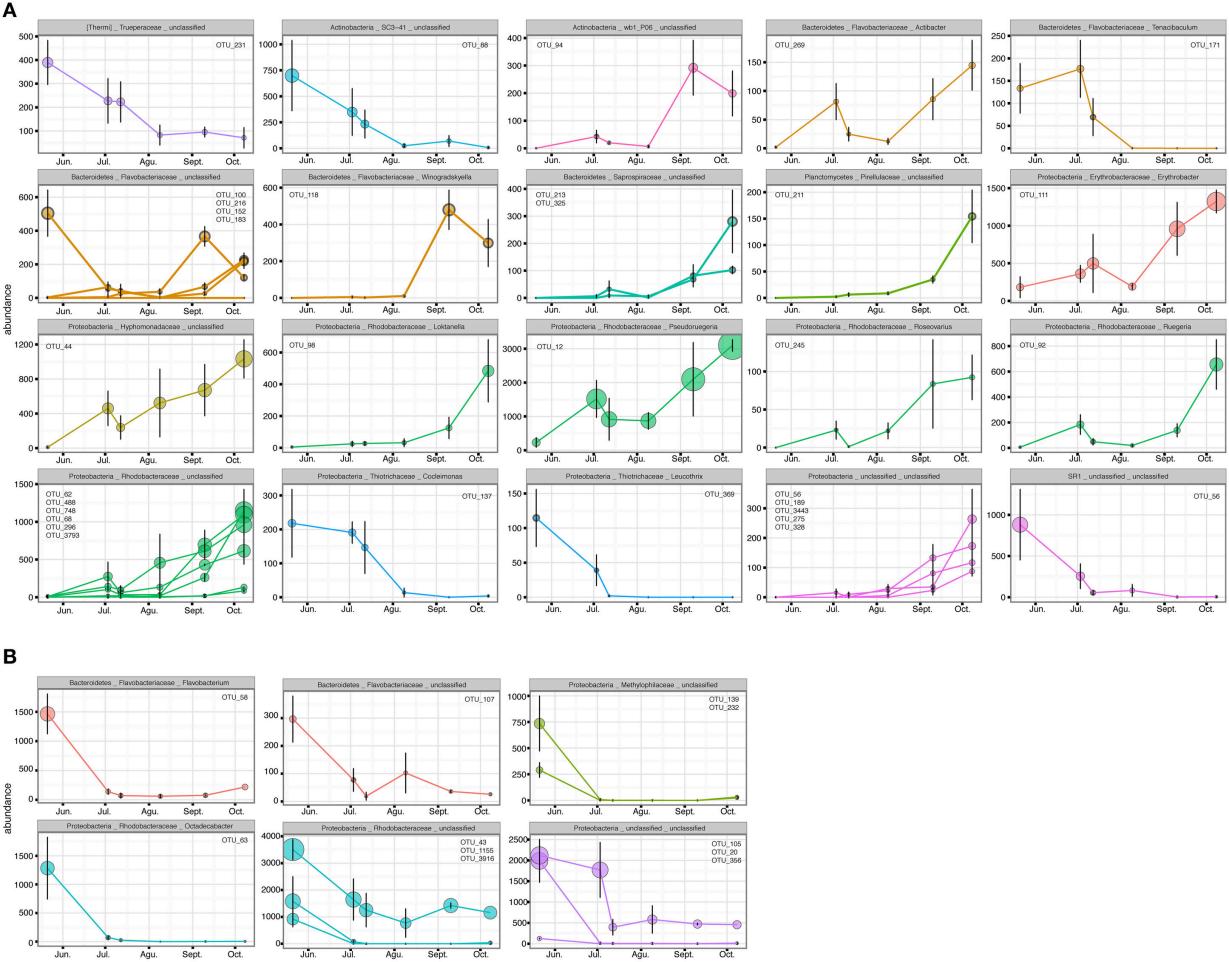
**OTUs with higher Pearson correlation (>0.6) in their abundance over time, contributing to the differences in the epiphytic bacteria of *C. compressa* (A) and surrounding seawater (B) between time 1 and 6 (May-October)**. The OTUs are grouped by phylum, family and genus. OTUs number are reported inside each graph.

The PERMANOVA revealed significant differences of bacterial community between *C. compressa* and surrounding seawater in all terms [Table S6; Habitats, pseudo-F_(**df** = 1, 17)_ = 16.459, *p* < 0.05; Date, pseudo-F_(**df** = 5, 17)_ = 2.0629 *p* < 0.05; Habitats × Date, pseudo-F_(**df** = 5, 17)_ = 1.9768, *p* < 0.05]. The PCoA ordination displayed these differences (Figure 6). The proportion of variance accounted for by the first two axes was 70.4%. This high value makes us confident that our interpretation of the first pair of axes extracts most relevant information from the data. The first axis accounted for the major part of the variance (61.9%) and highlights the big differences between seawater on the one hand and the thallus surface on the other hand (Figure 6). The second axis accounted the 8.5% of the total variation and reflects the time series. This axis revealed a clear successional pattern of the epiphytic bacterial community of *C. compressa* from May to October that was not observed in the seawater samples (Figure 6). The successional pattern in *C. compressa* was also reflected in a continuous increase of OTUs richness that conversely was not observed in the surrounding seawater (Figure 7). Of the 3227 OTUs, a subset of 400 represented 90% of the diversity of the epiphytic bacteria on *C. compressa*. Of these, 173 were present in all samples. SIMPER analysis revealed a high number of OTUs contributing both to the similarity between samples at the same time point as well as to differences between sampling times. Between May (t1) and October (t6) 102 OTUs contributed to 66.9% of dissimilarity (Table S7A). Of these, 32 OTUs showed higher Pearson correlation (>0.6) in their abundance over time with some OTUs that tend to decrease or increase from May to October (Figure 8A). In October we observed an increase of OTUs belonging to *Rhodobacteraceae*. In particular the genera *Ruegeria, Nautella, Aquimarina, Loktanella, Saprospira*, and *Phaeobacter* increased in abundance with the natural degradation of the thalli of *C. compressa* (Figure S2).

With respect to the seawater, of a total of 1085 OTUs, 100 represented 98% of the bacterial diversity. SIMPER analysis revealed that 27 OTUs contributed to the 32.6% of dissimilarity between May (t1) and October (t6) (Table S7B). Of these 11 OTUs decreased in their abundance from May to October (cor. >0.6) (Figure 8B). Two of these were identified at genus level as *Octadecabacter* (family *Rhodobacteraceae*) and *Flavobacterium* (family *Flavobacteriaceae*) (Table S7B). Finally, of the higher correlated OTUs found in the two habitats from May to October there were not shared taxa.

## Discussion

We describe for the first time the bacterial communities of the canopy-forming alga *Cystoseira compressa* and surrounding seawater using next generation sequencing data. The most abundant groups of bacteria in both habitats belonged to *Proteobacteria* and *Bacteroidetes*. Consistent with other studies (Staufenberger et al., 2008; Lachnit et al., 2009; Bengtsson et al., 2010; Burke et al., 2011), we found a clear difference between the bacterial communities of *C. compressa* and the surrounding seawater. The bacterial community of seawater remains more stable compared to that on *C. compressa*, that showed a clear successional pattern associated to aging thalli. These variations were characterized by gradual addition of OTUs (*Verrucomicrobia, Actinobacteria* and SR1) to the epiphytic community, suggesting a clear successional trend. We also found an increase in abundance of potential microbial pathogens associated to older thalli of *C. compressa*.

Even though biofilm-forming bacteria need to be recruited from the surrounding environment, the large differences between seaweed-associated bacteria and those of the surrounding water column are indicative of a selection process whereby the seaweed, the bacteria or a combination of both have the capacity to modulate the recruitment of the biofilm. Our results support the idea of the presence of generalist epiphytes common to all or many macroalgae (Egan et al., 2013). *Alphaproteobacteri*a, *Bacteroidetes, Cyanobacteria, Verrucomicrobia* were abundantly found on other brown algae such as *Fucus vesiculosus* (Lachnit et al., 2011), *Saccharina latissima* (Staufenberger et al., 2008) as well as green algae (Burke et al., 2011). Of the four most abundant OTUs detected in this study two were identified as *Loktanella* and *Pseudoruegeria*. Different species of *Loktanella* have been found on *Fucus vesiculosus* (Lachnit et al., 2011; Stratil et al., 2013), *Ulva australis* (Burke et al., 2011) and other macroalgal species (Egan et al., 2013; Hollants et al., 2013; Miranda et al., 2013). The presence of these genera can be related to their capacity to utilize organic carbon sources released from the seaweeds (Bengtsson et al., 2011). The latter provide substrate but also nutrients and trigger chemotactic behavior of bacteria that are highly adaptive and capable of rapid metabolization of algal exudates (Goecke et al., 2010; Wahl et al., 2012).

During spring (~May), when new branches of *C. compressa* provide a fresh substrate for colonizing bacteria, the epiphytic bacterial community was characterized by lower OTU richness, evenness and Shannon index. The low evenness was due a low number of OTUs and the dominance of 8 OTUs mainly belonging to the *Proteobacteria* that make up nearly 50% of the sequences in spring. We hypothesize that the lower OTU richness found in spring is a consequence of a natural colonization process of the microbial biofilm. In July, thalli of *C. compressa* at the study site reach their maximum dimension and physiological activity. Even though not directly observed in *C. compressa* species, the increase of seawater temperature induces a high photosynthetic activity and concomitant exudation rates of carbohydrates (Abdullah and Fredriksen, 2004; Wada et al., 2007) that can be beneficial for heterotrophic bacteria (Bengtsson et al., 2011, 2012). Hence, in July the growth of the epiphytic bacteria on *C. compressa* leads to an increase of OTUs richness and higher evenness values indicative of the presence of a well-structured community. The shift of the epiphytic community from May to July is also reflected by the increase of reads belonging to *Cyanobacteria*. This aspect was also observed on *Fucus vesiculosus* (Lachnit et al., 2011). In August, when *C. compressa* sheds the majority of upright annual axes, the epiphytic community of *C. compressa* seems to undergo important changes. In fact we observed a drastic decrease to half of OTU richness compared to July. However, we did not observe the same reduction on the evenness values. In September-October the OTUs richness and evenness seems to recover to levels observed in July. The higher OTUs richness may result from the decrease of the seaweed's physiological activity and antimicrobial activity whereby the quiescent status of the alga would explain the increase in abundances of *Rhodobacteraceae* and in particular of different genera such as *Ruegeria, Nautella, Aquimarina, Loktanella, Saprospira*, and *Phaeobacter* as already observed in bleached parts of the red seaweed *Delisea pulchra* (Case et al., 2011; Fernandes et al., 2011, 2012; Zozaya-Valdes et al., 2015).

Extensive loss of *Cystoseira* species, including *C. compressa*, has been reported in recent years, which has been attributed to the interacting effects of local and global stressors (Perkol-Finkel and Airoldi, 2010; Strain et al., 2015). The exact mechanisms behind these losses have not been fully understood yet, and ongoing experiments have led to the hypothesis of a possible, but up till now unexplored, role of the microbial communities. In fact, the tight interaction between bacteria and their host suggests that the epiphytic microbial community can play an important role in the resilience capability of their host. Moreover, the metabolic capability of bacteria to grow and divide very rapidly may result in bacteria responding faster to external stressors compared their host. In this perspective bacteria should be a potential first indicator of environmental or anthropogenic stressors. Our results provide an important base-knowledge as first step to analyze the possible mechanisms by which *Cystoseira* interacts with surface bacteria. In fact, understanding the temporal dynamics of epiphytic bacteria under natural conditions can help to identify possible modifications of the biofilm due to external factors of stress. Then experiments should be performed to explore the response of the holobiont under the combined effects of local and global stressors known to be major causes of the loss of *C. compressa*. Particular consideration should be given to those taxa, such as *Ruegeria, Nautella, Aquimarina, Loktanella, Saprospira*, and *Phaeobacter*, that tend to be more present during the natural degradation of *C. compressa*, to observe if stressors can directly increase the abundance of these taxa or alternatively affect the antimicrobial activities of the seaweed with consequent rise of deleterious taxa.

## Author contributions

FM, LA, OD, and AW designed the work. FM Acquisition, analysis and interpretation of data for the work. SD assisted FM with the molecular lab work. All authors contributed to the writing of the paper.

### Conflict of interest statement

The authors declare that the research was conducted in the absence of any commercial or financial relationships that could be construed as a potential conflict of interest.
