# Supplementary material for: Diversity and Temporal Dynamics of the Epiphytic Bacterial Communities Associated with the Canopy-Forming Seaweed Cystoseira compressa (Esper) Gerloff and Nizamuddin
Source: Front Microbiol. 2016 Apr 8;7:476. doi: 10.3389/fmicb.2016.00476 (PMC4824759; doi:10.3389/fmicb.2016.00476)
Supplement: Supplementary file 1 [file Table1.DOCX]

Supplementary Material

**Diversity and temporal dynamics of the epiphytic bacterial communities associated with the canopy-forming seaweed *Cystoseira compressa* (Esper) Gerloff & Nizamuddin**

**Francesco Paolo Mancuso^*^, Sofie D'hondt, Anne Willems, Laura Airoldi^*^ and Olivier De Clerck**

***Correspondence:** Francesco Paolo Mancuso, Dipartimento di Scienze Biologiche, Geologiche ed Ambientali, University of Bologna, via Sant'Alberto 163, Ravenna, 48123, Italy.

francesco.mancuso4@unibo.it

Laura Airoldi, Dipartimento di Scienze Biologiche, Geologiche ed Ambientali, University of Bologna, via Sant'Alberto 163, Ravenna, 48123, Italy.

laura.airoldi@unibo.it

# Supplementary Table

**Table S1.** Details of the number of reads per sample retained after different quality filtering steps. Sample 73 was omitted from the analyses due to the low number of reads.

| **Substrate** | **ID** | **Date** | **Merged** | **Filtered** | **removed singleton and chimeras** | **removed chlorophast, mithocondria, less than 1000 reads, removed Oscillatoriophycideae and Synechococcophycideae OTUs** |
| --- | --- | --- | --- | --- | --- | --- |
| *C.compressa* | 8 | 21-05-2014 | 119,823 | 44,637 | 33,250 | 31,672 |
| *C.compressa* | 9 | 21-05-2014 | 112,486 | 40,601 | 33,422 | 23,533 |
| *C.compressa* | 10 | 21-05-2014 | 172,598 | 76,296 | 64,945 | 40,367 |
| seawater | 12 | 21-05-2014 | 164,537 | 58,083 | 44,742 | 43,263 |
| seawater | 13 | 21-05-2014 | 204,201 | 86,643 | 70,293 | 68,432 |
| *C.compressa* | 2 | 03-07-2014 | 213,826 | 87,441 | 73,397 | 63,373 |
| *C.compressa* | 4 | 03.07.2014 | 306,453 | 87,680 | 73,808 | 68,352 |
| *C.compressa* | 14 | 03-07-2014 | 234,800 | 90,948 | 80,918 | 75,903 |
| seawater | 15 | 03-07-2014 | 271,811 | 116,017 | 69,345 | 65,393 |
| seawater | 16 | 03-07-2014 | 198,640 | 83,520 | 76,064 | 69,724 |
| *C.compressa* | 17 | 12-07-2014 | 105,541 | 37,160 | 30,435 | 26,479 |
| *C.compressa* | 18 | 12-07-2014 | 202,423 | 85,283 | 56,808 | 49,302 |
| *C.compressa* | 19 | 12-07-2014 | 169,705 | 66,655 | 57,166 | 51,435 |
| seawater | 31 | 12-07-2014 | 83,195 | 18,172 | 14,074 | 12,157 |
| seawater | 32 | 12-07-2014 | 152,240 | 66,933 | 58,861 | 54,799 |
| *C.compressa* | 23 | 09-08-2014 | 75,813 | 28,960 | 23,538 | 20,605 |
| *C.compressa* | 24 | 09-08-2014 | 115,872 | 44,243 | 35,506 | 33,153 |
| *C.compressa* | 25 | 09-08-2014 | 85,123 | 20,557 | 8,878 | 8,727 |
| seawater | 29 | 09-08-2014 | 83,669 | 29,721 | 26,816 | 22,393 |
| seawater | 30 | 09-08-2014 | 67,786 | 29,403 | 17,081 | 16,156 |
| *C.compressa* | 47 | 10-09-2014 | 156,830 | 57,344 | 43,053 | 41,304 |
| *C.compressa* | 48 | 10-09-2014 | 156,693 | 57,094 | 49,485 | 46,930 |
| *C.compressa* | 49 | 10-09-2014 | 190,610 | 68,437 | 53,770 | 46,105 |
| seawater | 59 | 10-09-2014 | 156,220 | 64,291 | 51,538 | 45,470 |
| seawater | 60 | 10-09-2014 | 168,793 | 53,840 | 41,601 | 37,125 |
| *C.compressa* | 61 | 08-10-2014 | 207,731 | 79,571 | 65,721 | 55,338 |
| *C.compressa* | 62 | 08-10-2014 | 258,970 | 105,155 | 81,269 | 73,531 |
| *C.compressa* | 63 | 08-10-2014 | 234,040 | 89,127 | 62,008 | 52,993 |
| seawater | 73 | 08-10-2014 | 60,354 | 343 | 113 | 0 |
| seawater | 74 | 08-10-2014 | 185,930 | 63,595 | 47,635 | 45,585 |
| negative sample | 75 |  | 10,525 | 2,875 | 2,075 | 0 |
|  | **Tot.** |  | **4,927,238** | **1,840,625** | **1,447,615** | **1,289,599** |
