# Supplementary material for: Diversity and Temporal Dynamics of the Epiphytic Bacterial Communities Associated with the Canopy-Forming Seaweed Cystoseira compressa (Esper) Gerloff and Nizamuddin
Source: Front Microbiol. 2016 Apr 8;7:476. doi: 10.3389/fmicb.2016.00476 (PMC4824759; doi:10.3389/fmicb.2016.00476)
Supplement: Supplementary file 2 [file Table2.DOCX]

Supplementary Material

**Diversity and temporal dynamics of the epiphytic bacterial communities associated with the canopy-forming seaweed *Cystoseira compressa* (Esper) Gerloff & Nizamuddin**

**Francesco Paolo Mancuso^*^, Sofie D'hondt, Anne Willems, Laura Airoldi^*^ and Olivier De Clerck**

***Correspondence:** Francesco Paolo Mancuso, Dipartimento di Scienze Biologiche, Geologiche ed Ambientali, University of Bologna, via Sant'Alberto 163, Ravenna, 48123, Italy.

francesco.mancuso4@unibo.it

Laura Airoldi, Dipartimento di Scienze Biologiche, Geologiche ed Ambientali, University of Bologna, via Sant'Alberto 163, Ravenna, 48123, Italy.

laura.airoldi@unibo.it

# Supplementary Table

**Table S2.** Summary statistics of the number of reads of *C. compressa* and surrounding seawater: number of samples, taxa and total, mean, standard deviation, minimum and maximum number of reads.

| **Substrate** | **n° samples** | **taxa** | **Total seqs.** | **mean** | **min** | **max** | **sd** |
| --- | --- | --- | --- | --- | --- | --- | --- |
| all samples | 29 | 3820 | 1,289.559 | 44.469 | 8.727 | 75.903 | 18.999 |
| *C. compressa* | 18 | 3227 | 809.102 | 44.950 | 8.727 | 75.903 | 18.717 |
| Seawater | 11 | 1085 | 480.497 | 43.682 | 12.157 | 69.724 | 20.348 |
