# Supplementary material for: Diversity and Temporal Dynamics of the Epiphytic Bacterial Communities Associated with the Canopy-Forming Seaweed Cystoseira compressa (Esper) Gerloff and Nizamuddin
Source: Front Microbiol. 2016 Apr 8;7:476. doi: 10.3389/fmicb.2016.00476 (PMC4824759; doi:10.3389/fmicb.2016.00476)
Supplement: Supplementary file 3 [file Table3.DOCX]

Supplementary Material

**Diversity and temporal dynamics of the epiphytic bacterial communities associated with the canopy-forming seaweed *Cystoseira compressa* (Esper) Gerloff & Nizamuddin**

**Francesco Paolo Mancuso^*^, Sofie D'hondt, Anne Willems, Laura Airoldi^*^ and Olivier De Clerck**

***Correspondence:** Francesco Paolo Mancuso, Dipartimento di Scienze Biologiche, Geologiche ed Ambientali, University of Bologna, via Sant'Alberto 163, Ravenna, 48123, Italy.

francesco.mancuso4@unibo.it

Laura Airoldi, Dipartimento di Scienze Biologiche, Geologiche ed Ambientali, University of Bologna, via Sant'Alberto 163, Ravenna, 48123, Italy.

laura.airoldi@unibo.it

# Supplementary Table

**Table S3.** PERMANOVA results of alpha diversity indices.

| Resemblance: D1 Euclidean distance | | |  |  |  |  |
| --- | --- | --- | --- | --- | --- | --- |
| Sums of squares type: Type III (partial) | | |  |  |  |  |
| Fixed effects sum to zero for mixed terms | | | |  |  |  |
| Permutation method: Unrestricted permutation of raw data | | | | |  |  |
| Number of permutations: 9999 | | |  |  |  |  |
|  |  |  |  |  |  |  |
| Factors |  |  |  |  |  |  |
| Name | Abbrev. | Type | Levels |  |  |  |
| Habitat | Ha | Fixed | 2 |  |  |  |
| Date | Da | Random | 6 |  |  |  |
|  |  |  |  |  |  |  |
| PERMANOVA table of results **OTU_richness** | | | |  |  |  |
|  |  |  |  |  |  | Unique |
| Source | df | SS | MS | Pseudo-F | P(perm) | perms |
| Ha | 1 | 7.63E+05 | 7.63E+05 | 67.886 | 0.0002 | 9806 |
| Da | 5 | 1.66E+05 | 33150 | 4.1378 | 0.0113 | 9943 |
| HaxDa | 5 | 56654 | 11331 | 1.4143 | 0.2605 | 9966 |
| Res | 17 | 1.36E+05 | 8011.5 |  |  |  |
| Total | 28 | 1.23E+06 |  |  |  |  |
|  |  |  |  |  |  |  |
|  |  |  |  |  |  |  |
| PERMANOVA table of results **Shannon** | | |  |  |  |  |
|  |  |  |  |  |  | Unique |
| Source | df | SS | MS | Pseudo-F | P(perm) | perms |
| Ha | 1 | 18.628 | 18.628 | 50.953 | 0.0017 | 9844 |
| Da | 5 | 1.334 | 0.2668 | 1.7747 | 0.1734 | 9955 |
| HaxDa | 5 | 1.8567 | 0.37134 | 2.47 | 0.067 | 9962 |
| Res | 17 | 2.5557 | 0.15034 |  |  |  |
| Total | 28 | 26.172 |  |  |  |  |
|  |  |  |  |  |  |  |
|  |  |  |  |  |  |  |
| PERMANOVA table of results **evenness** | | | |  |  |  |
|  |  |  |  |  |  | Unique |
| Source | df | SS | MS | Pseudo-F | P(perm) | perms |
| Ha | 1 | 0.16833 | 0.16833 | 18.028 | 0.0116 | 9760 |
| Da | 5 | 1.73E-02 | 3.46E-03 | 0.90747 | 0.5027 | 9968 |
| HaxDa | 5 | 4.74E-02 | 9.48E-03 | 2.4873 | 0.069 | 9947 |
| Res | 17 | 6.48E-02 | 3.81E-03 |  |  |  |
| Total | 28 | 0.30574 |  |  |  |  |
|  |  |  |  |  |  |  |
|  |  |  |  |  |  |  |
| PERMANOVA table of results **Chao1** | | |  |  |  |  |
|  |  |  |  |  |  | Unique |
| Source | df | SS | MS | Pseudo-F | P(perm) | perms |
| Ha | 1 | 1.01E+06 | 1.01E+06 | 104.84 | 0.0001 | 9824 |
| Da | 5 | 3.17E+05 | 63401 | 3.2481 | 0.0308 | 9940 |
| HaxDa | 5 | 46963 | 9392.5 | 0.48118 | 0.787 | 9954 |
| Res | 17 | 3.32E+05 | 19520 |  |  |  |
| Total | 28 | 1.84E+06 |  |  |  |  |
