# Supplementary material for: Diversity and Temporal Dynamics of the Epiphytic Bacterial Communities Associated with the Canopy-Forming Seaweed Cystoseira compressa (Esper) Gerloff and Nizamuddin
Source: Front Microbiol. 2016 Apr 8;7:476. doi: 10.3389/fmicb.2016.00476 (PMC4824759; doi:10.3389/fmicb.2016.00476)
Supplement: Supplementary file 4 [file Table4.DOCX]

Supplementary Material

**Diversity and temporal dynamics of the epiphytic bacterial communities associated with the canopy-forming seaweed *Cystoseira compressa* (Esper) Gerloff & Nizamuddin**

**Francesco Paolo Mancuso^*^, Sofie D'hondt, Anne Willems, Laura Airoldi^*^ and Olivier De Clerck**

***Correspondence:** Francesco Paolo Mancuso, Dipartimento di Scienze Biologiche, Geologiche ed Ambientali, University of Bologna, via Sant'Alberto 163, Ravenna, 48123, Italy.

francesco.mancuso4@unibo.it

Laura Airoldi, Dipartimento di Scienze Biologiche, Geologiche ed Ambientali, University of Bologna, via Sant'Alberto 163, Ravenna, 48123, Italy.

laura.airoldi@unibo.it

# Supplementary Table

**Table S4.** Relative abundance of the top 300 OTU at the phylum level on *C. compressa* and surrounding seawater. Values are percentages. Mean= overall mean per phylum.

|  | **May** | | **July** | | | | **August** | | **September** | | **October** | |  |
| --- | --- | --- | --- | --- | --- | --- | --- | --- | --- | --- | --- | --- | --- |
|  | 21/05/14 | | 03/07/14 | | 12/07/14 | | 09/08/14 | | 10/09/14 | | 08/10/14 | |  |
|  | *C. compressa* | seawater | *C. compressa* | Seawater | *C. compressa* | seawater | *C. compressa* | seawater | *C. compressa* | seawater | *C. compressa* | seawater | **mean** |
| **Proteobacteria** | 44.8 | 89.6 | 44.2 | 91.5 | 43.6 | 92.1 | 55.7 | 89.5 | 55.4 | 80.8 | 60.3 | 88.4 | 69.7 |
| **Bacteroidetes** | 18.7 | 5.9 | 14.4 | 4.6 | 10.1 | 4.5 | 12.2 | 6.1 | 10.0 | 15.8 | 8.5 | 5.8 | 9.7 |
| **Actinobacteria** | 5.3 | 1.0 | 8.0 | 0.4 | 7.7 | 0.3 | 4.6 | 1.1 | 2.8 | 0.6 | 2.0 | 1.4 | 2.9 |
| **Verrucomicrobia** | 12.7 | 0.1 | 3.8 | 0.1 | 4.3 | 0.1 | 4.3 | 0.2 | 2.7 | 0.1 | 3.5 | 0.5 | 2.7 |
| **Cyanobacteria** | 1.0 | 0.0 | 4.4 | 0.0 | 12.5 | 0.1 | 3.1 | 0.0 | 4.4 | 0.0 | 1.0 | 0.0 | 2.2 |
| **SR1** | 4.6 | 0.0 | 0.6 | 0.0 | 0.4 | 0.0 | 0.6 | 0.0 | 0.8 | 0.0 | 0.0 | 0.0 | 0.6 |
| **GN02** | 2.7 | 0.0 | 0.2 | 0.0 | 0.6 | 0.0 | 0.0 | 0.0 | 0.3 | 0.0 | 1.7 | 0.0 | 0.5 |
| **OD1** | 1.4 | 0.1 | 1.3 | 0.0 | 0.5 | 0.0 | 1.7 | 0.0 | 0.2 | 0.0 | 0.2 | 0.0 | 0.5 |
| **Chloroflexi** | 0.1 | 0.0 | 0.8 | 0.0 | 1.6 | 0.0 | 2.5 | 0.0 | 0.2 | 0.0 | 0.2 | 0.0 | 0.4 |
| **[Thermi]** | 1.3 | 0.0 | 0.4 | 0.0 | 0.6 | 0.0 | 2.0 | 0.0 | 0.4 | 0.0 | 0.2 | 0.0 | 0.4 |
| **Planctomycetes** | 0.2 | 0.0 | 0.3 | 0.0 | 0.5 | 0.0 | 0.5 | 0.0 | 0.7 | 0.0 | 0.5 | 0.0 | 0.2 |
| **unclassified** | 0.0 | 0.0 | 0.2 | 0.0 | 0.2 | 0.0 | 0.3 | 0.0 | 0.9 | 0.0 | 0.0 | 0.0 | 0.1 |
| **Fusobacteria** | 0.0 | 0.0 | 0.1 | 0.0 | 0.3 | 0.0 | 0.2 | 0.0 | 0.1 | 0.0 | 1.0 | 0.0 | 0.1 |
| **TM7** | 1.0 | 0.0 | 0.1 | 0.0 | 0.2 | 0.0 | 0.1 | 0.0 | 0.0 | 0.0 | 0.0 | 0.0 | 0.1 |
