# Supplementary material for: Diversity and Temporal Dynamics of the Epiphytic Bacterial Communities Associated with the Canopy-Forming Seaweed Cystoseira compressa (Esper) Gerloff and Nizamuddin
Source: Front Microbiol. 2016 Apr 8;7:476. doi: 10.3389/fmicb.2016.00476 (PMC4824759; doi:10.3389/fmicb.2016.00476)
Supplement: Supplementary file 5 [file Table5.DOCX]

Supplementary Material

**Diversity and temporal dynamics of the epiphytic bacterial communities associated with the canopy-forming seaweed *Cystoseira compressa* (Esper) Gerloff & Nizamuddin**

**Francesco Paolo Mancuso^*^, Sofie D'hondt, Anne Willems, Laura Airoldi^*^ and Olivier De Clerck**

***Correspondence:** Francesco Paolo Mancuso, Dipartimento di Scienze Biologiche, Geologiche ed Ambientali, University of Bologna, via Sant'Alberto 163, Ravenna, 48123, Italy.

francesco.mancuso4@unibo.it

Laura Airoldi, Dipartimento di Scienze Biologiche, Geologiche ed Ambientali, University of Bologna, via Sant'Alberto 163, Ravenna, 48123, Italy.

laura.airoldi@unibo.it

# Supplementary Table

**Table S5.** Relative abundance of the top 300 OTU at the family level on *C. compressa* and surrounding seawater. Values are percentages. Mean= overall mean per family per habitat.

|  | **May** | | **July** | | | | **August** | | **September** | | **October** | |  |  |
| --- | --- | --- | --- | --- | --- | --- | --- | --- | --- | --- | --- | --- | --- | --- |
|  | 21/05/14 | | 03/07/14 | | 12/07/14 | | 09/08/14 | | 10/09/14 | | 08/10/14 | | **mean** | **mean** |
|  | *C. compressa* | seawater | *C. compressa* | seawater | *C. compressa* | seawater | *C. compressa* | seawater | *C. compressa* | seawater | *C. compressa* | seawater | *C. compressa* | seawater |
| Rhodobacteraceae | 31.6 | 19.6 | 27.2 | 27.0 | 27.0 | 36.0 | 39.8 | 18.2 | 39.9 | 47.0 | 42.7 | 18.0 | 34.7 | 27.6 |
| Pelagibacteraceae | 0.0 | 43.9 | 0.4 | 42.8 | 0.2 | 34.1 | 0.1 | 51.3 | 0.1 | 19.4 | 0.0 | 49.7 | 0.1 | 40.2 |
| unclassified | 13.5 | 22.3 | 17.2 | 15.0 | 24.2 | 16.3 | 16.6 | 15.4 | 13.3 | 11.4 | 8.2 | 16.2 | 15.5 | 16.1 |
| Flavobacteriaceae | 11.7 | 5.8 | 5.9 | 2.5 | 5.6 | 2.9 | 6.2 | 4.2 | 4.7 | 2.7 | 5.8 | 4.1 | 6.6 | 3.7 |
| Verrucomicrobiaceae | 12.7 | 0.0 | 3.8 | 0.0 | 4.3 | 0.0 | 4.3 | 0.0 | 2.7 | 0.0 | 3.5 | 0.0 | 5.2 | 0.0 |
| Saprospiraceae | 7.0 | 0.0 | 6.8 | 0.0 | 4.2 | 0.1 | 5.6 | 0.0 | 5.1 | 0.0 | 2.3 | 0.0 | 5.2 | 0.0 |
| Phyllobacteriaceae | 8.0 | 0.1 | 4.4 | 0.0 | 5.4 | 0.0 | 3.7 | 0.0 | 3.2 | 0.0 | 2.5 | 0.0 | 4.6 | 0.0 |
| Erythrobacteraceae | 0.5 | 0.8 | 1.3 | 2.3 | 1.5 | 3.0 | 1.9 | 0.1 | 3.0 | 1.2 | 3.1 | 0.3 | 1.9 | 1.3 |
| Thiotrichaceae | 1.9 | 0.0 | 3.2 | 0.0 | 2.9 | 0.0 | 1.3 | 0.0 | 1.4 | 0.0 | 4.6 | 0.0 | 2.5 | 0.0 |
| Cryomorphaceae | 0.0 | 0.0 | 0.0 | 0.1 | 0.0 | 0.7 | 0.0 | 0.1 | 0.0 | 11.8 | 0.0 | 0.0 | 0.0 | 2.1 |
| Hyphomonadaceae | 1.1 | 0.0 | 1.7 | 0.1 | 1.8 | 0.0 | 3.0 | 0.0 | 2.4 | 0.0 | 2.3 | 0.0 | 2.1 | 0.0 |
| SC3-41 | 3.5 | 0.0 | 2.8 | 0.0 | 2.2 | 0.0 | 0.8 | 0.0 | 0.5 | 0.0 | 0.9 | 0.0 | 1.8 | 0.0 |
| Halomonadaceae | 0.0 | 0.3 | 0.0 | 1.1 | 0.0 | 1.6 | 0.0 | 3.5 | 0.0 | 0.7 | 0.0 | 2.1 | 0.0 | 1.6 |
| [Balneolaceae] | 0.0 | 0.1 | 0.0 | 2.1 | 0.0 | 0.8 | 0.0 | 1.8 | 0.0 | 1.3 | 0.0 | 1.7 | 0.0 | 1.3 |
| Trueperaceae | 1.3 | 0.0 | 0.4 | 0.0 | 0.6 | 0.0 | 2.0 | 0.0 | 0.4 | 0.0 | 0.2 | 0.0 | 0.8 | 0.0 |
