# Supplementary material for: Diversity and Temporal Dynamics of the Epiphytic Bacterial Communities Associated with the Canopy-Forming Seaweed Cystoseira compressa (Esper) Gerloff and Nizamuddin
Source: Front Microbiol. 2016 Apr 8;7:476. doi: 10.3389/fmicb.2016.00476 (PMC4824759; doi:10.3389/fmicb.2016.00476)
Supplement: Supplementary file 6 [file Table6.DOCX]

| Resemblance: S17 Bray Curtis similarity (+d) | | | |  |  |  |
| --- | --- | --- | --- | --- | --- | --- |
| Sums of squares type: Type III (partial) | | |  |  |  |  |
| Fixed effects sum to zero for mixed terms | | | |  |  |  |
| Permutation method: Unrestricted permutation of raw data | | | | |  |  |
| Number of permutations: 9999 | | |  |  |  |  |
|  |  |  |  |  |  |  |
| Factors |  |  |  |  |  |  |
| Name | Abbrev. | Type | Levels |  |  |  |
| Habitat | Ha | Fixed | 2 |  |  |  |
| Date | Da | Random | 6 |  |  |  |
|  |  |  |  |  |  |  |
| PERMANOVA table of results **Beta diversity of *C. compressa* and surrounding seawater** | | | | | | |
|  |  |  |  |  |  | Unique |
| Source | df | SS | MS | Pseudo-F | P(perm) | perms |
| **Ha** | 1 | 39261 | 39261 | 16.459 | **0.0006** | 9911 |
| **Da** | 5 | 12608 | 2521.6 | 2.0629 | **0.0104** | 9904 |
| **HaxDa** | 5 | 12082 | 2416.4 | 1.9768 | **0.0165** | 9890 |
| Res | 17 | 20780 | 1222.4 |  |  |  |
| Total | 28 | 89291 |  |  |  |  |

Supplementary Material

**Diversity and temporal dynamics of the epiphytic bacterial communities associated with the canopy-forming seaweed *Cystoseira compressa* (Esper) Gerloff & Nizamuddin**

**Francesco Paolo Mancuso^*^, Sofie D'hondt, Anne Willems, Laura Airoldi^*^ and Olivier De Clerck**

***Correspondence:** Francesco Paolo Mancuso, Dipartimento di Scienze Biologiche, Geologiche ed Ambientali, University of Bologna, via Sant'Alberto 163, Ravenna, 48123, Italy.

francesco.mancuso4@unibo.it

Laura Airoldi, Dipartimento di Scienze Biologiche, Geologiche ed Ambientali, University of Bologna, via Sant'Alberto 163, Ravenna, 48123, Italy.

laura.airoldi@unibo.it

# Supplementary Table

**Table S6.** Results of the PERMANOVA analysis, testing for significant differences in bacterial composition as a function of habitat (Ha) and sampling time (Da).
