# Supplementary material for: Diversity and Temporal Dynamics of the Epiphytic Bacterial Communities Associated with the Canopy-Forming Seaweed Cystoseira compressa (Esper) Gerloff and Nizamuddin
Source: Front Microbiol. 2016 Apr 8;7:476. doi: 10.3389/fmicb.2016.00476 (PMC4824759; doi:10.3389/fmicb.2016.00476)
Supplement: Supplementary file 7 [file Table7.DOCX]

Supplementary Material

**Diversity and temporal dynamics of the epiphytic bacterial communities associated with the canopy-forming seaweed *Cystoseira compressa* (Esper) Gerloff & Nizamuddin**

**Francesco Paolo Mancuso^*^, Sofie D'hondt, Anne Willems, Laura Airoldi^*^ and Olivier De Clerck**

***Correspondence:** Francesco Paolo Mancuso, Dipartimento di Scienze Biologiche, Geologiche ed Ambientali, University of Bologna, via Sant'Alberto 163, Ravenna, 48123, Italy.

francesco.mancuso4@unibo.it

Laura Airoldi, Dipartimento di Scienze Biologiche, Geologiche ed Ambientali, University of Bologna, via Sant'Alberto 163, Ravenna, 48123, Italy.

laura.airoldi@unibo.it

# Supplementary Table

**Table S7.** Results of the SIMPER analysis of the epiphytic bacteria of *C. compressa* (A) and surrounding seawater (B). OTUs contributed to the differences between time 1 (May) and time 6 (October).

| **(A) SIMPER analysis epiphytic bacteria of *C. compressa*** | | | | | | | | | | | | | | | |
| --- | --- | --- | --- | --- | --- | --- | --- | --- | --- | --- | --- | --- | --- | --- | --- |
| Groups t1 & t6 |  |  |  | | |  | |  |  |  |  |  |  |  |  |
| Average dissimilarity = 66.94 | | | | | |  | |  |  |  |  |  |  |  |  |
|  | Group t1 | Group t6 |  | |  | |  | |  |  |  |  |  |  |  |
| Species | Av.Abund | Av.Abund | Av.Diss | Diss/SD | | | Contrib% | | Cum.% | Kingdom | Phylum | Class | Order | Family | Genus |
| OTU_8 | 26.17 | 72.2 | 1.14 | 1.03 | | | 1.7 | | 1.7 | Bacteria | Proteobacteria | Alphaproteobacteria | Rhodobacterales | Rhodobacteraceae | unclassified |
| OTU_12 | 12.96 | 55.61 | 1.09 | 3.75 | | | 1.63 | | 3.34 | Bacteria | Proteobacteria | Alphaproteobacteria | Rhodobacterales | Rhodobacteraceae | Pseudoruegeria |
| OTU_29 | 12.16 | 51.23 | 0.98 | 6.17 | | | 1.47 | | 4.8 | Bacteria | Proteobacteria | Gammaproteobacteria | Thiotrichales | Thiotrichaceae | Thiothrix |
| OTU_18 | 38.24 | 2.79 | 0.89 | 2.14 | | | 1.33 | | 6.13 | Bacteria | Bacteroidetes | Flavobacteriia | Flavobacteriales | Flavobacteriaceae | Krokinobacter |
| OTU_22 | 31.73 | 0 | 0.82 | 1.47 | | | 1.23 | | 7.36 | Bacteria | Verrucomicrobia | Verrucomicrobiae | Verrucomicrobiales | Verrucomicrobiaceae | Haloferula |
| OTU_62 | 1.32 | 33.49 | 0.82 | 4.9 | | | 1.22 | | 8.58 | Bacteria | Proteobacteria | Alphaproteobacteria | Rhodobacterales | Rhodobacteraceae | unclassified |
| OTU_488 | 1.55 | 33.03 | 0.8 | 9.24 | | | 1.19 | | 9.77 | Bacteria | Proteobacteria | Alphaproteobacteria | Rhodobacterales | Rhodobacteraceae | unclassified |
| OTU_44 | 3.08 | 31.79 | 0.72 | 6.28 | | | 1.08 | | 10.86 | Bacteria | Proteobacteria | Alphaproteobacteria | Rhodobacterales | Hyphomonadaceae | unclassified |
| OTU_748 | 3.59 | 30.76 | 0.69 | 6.22 | | | 1.03 | | 11.88 | Bacteria | Proteobacteria | Alphaproteobacteria | Rhodobacterales | Rhodobacteraceae | unclassified |
| OTU_17 | 36.84 | 10.49 | 0.66 | 1.59 | | | 0.99 | | 12.87 | Bacteria | Verrucomicrobia | Verrucomicrobiae | Verrucomicrobiales | Verrucomicrobiaceae | Haloferula |
| OTU_11 | 51.47 | 24.63 | 0.65 | 0.88 | | | 0.97 | | 13.84 | Bacteria | Proteobacteria | Alphaproteobacteria | Rhodobacterales | Rhodobacteraceae | unclassified |
| OTU_4 | 66.69 | 45.96 | 0.64 | 1.3 | | | 0.96 | | 14.8 | Bacteria | Proteobacteria | Alphaproteobacteria | Rhodobacterales | Rhodobacteraceae | Loktanella |
| OTU_111 | 11.19 | 36.24 | 0.64 | 2.61 | | | 0.96 | | 15.76 | Bacteria | Proteobacteria | Alphaproteobacteria | Sphingomonadales | Erythrobacteraceae | Erythrobacter |
| OTU_56 | 25.6 | 1.33 | 0.63 | 1.49 | | | 0.94 | | 16.71 | Bacteria | SR1 | unclassified | unclassified | unclassified | unclassified |
| OTU_92 | 1.92 | 24.99 | 0.59 | 3.4 | | | 0.88 | | 17.59 | Bacteria | Proteobacteria | Alphaproteobacteria | Rhodobacterales | Rhodobacteraceae | Ruegeria |
| OTU_45 | 13.56 | 36.7 | 0.59 | 1.96 | | | 0.87 | | 18.46 | Bacteria | Proteobacteria | Alphaproteobacteria | Rhodobacterales | Rhodobacteraceae | Nautella |
| OTU_88 | 23.98 | 1.56 | 0.58 | 1.82 | | | 0.86 | | 19.32 | Bacteria | Actinobacteria | Acidimicrobiia | Acidimicrobiales | SC3-41 | unclassified |
| OTU_100 | 22 | 0 | 0.56 | 3.85 | | | 0.84 | | 20.16 | Bacteria | Bacteroidetes | Flavobacteriia | Flavobacteriales | Flavobacteriaceae | unclassified |
| OTU_68 | 2.63 | 24.3 | 0.55 | 3.94 | | | 0.82 | | 20.98 | Bacteria | Proteobacteria | Alphaproteobacteria | Rhodobacterales | Rhodobacteraceae | unclassified |
| OTU_91 | 20.77 | 0 | 0.53 | 1.29 | | | 0.8 | | 21.78 | Bacteria | GN02 | BD1-5 | unclassified | unclassified | unclassified |
| OTU_54 | 0.91 | 20.45 | 0.53 | 0.91 | | | 0.79 | | 22.57 | Bacteria | GN02 | unclassified | unclassified | unclassified | unclassified |
| OTU_138 | 2.92 | 22.89 | 0.51 | 2.19 | | | 0.77 | | 23.34 | Bacteria | Fusobacteria | Fusobacteriia | Fusobacteriales | Fusobacteriaceae | Cetobacterium |
| OTU_64 | 19.67 | 0 | 0.51 | 1.86 | | | 0.76 | | 24.1 | Bacteria | Bacteroidetes | [Saprospirae] | [Saprospirales] | Saprospiraceae | unclassified |
| OTU_59 | 0 | 20.28 | 0.51 | 1.35 | | | 0.75 | | 24.85 | Bacteria | Bacteroidetes | Flavobacteriia | Flavobacteriales | Flavobacteriaceae | Aquimarina |
| OTU_98 | 1.96 | 21.03 | 0.48 | 2.82 | | | 0.72 | | 25.57 | Bacteria | Proteobacteria | Alphaproteobacteria | Rhodobacterales | Rhodobacteraceae | Loktanella |
| OTU_25 | 27.18 | 8.78 | 0.46 | 2.18 | | | 0.69 | | 26.27 | Bacteria | Proteobacteria | Alphaproteobacteria | Rhizobiales | Phyllobacteriaceae | unclassified |
| OTU_6 | 4.43 | 22.82 | 0.46 | 2.63 | | | 0.69 | | 26.96 | Bacteria | Proteobacteria | Alphaproteobacteria | Sphingomonadales | Erythrobacteraceae | Erythrobacter |
| OTU_1371 | 10.01 | 27.53 | 0.46 | 1.46 | | | 0.69 | | 27.65 | Bacteria | Proteobacteria | Alphaproteobacteria | Rhodobacterales | Rhodobacteraceae | unclassified |
| OTU_93 | 0 | 17.71 | 0.44 | 1.26 | | | 0.66 | | 28.3 | Bacteria | Bacteroidetes | Flavobacteriia | Flavobacteriales | Flavobacteriaceae | unclassified |
| OTU_97 | 0 | 16.31 | 0.43 | 1.19 | | | 0.64 | | 28.94 | Bacteria | Bacteroidetes | [Saprospirae] | [Saprospirales] | Saprospiraceae | Saprospira |
| OTU_15 | 37.2 | 21.15 | 0.42 | 1.33 | | | 0.63 | | 29.57 | Bacteria | Proteobacteria | Alphaproteobacteria | Rhizobiales | Phyllobacteriaceae | Ahrensia |
| OTU_21 | 22.31 | 26.54 | 0.42 | 1.22 | | | 0.63 | | 30.2 | Bacteria | Verrucomicrobia | Verrucomicrobiae | Verrucomicrobiales | Verrucomicrobiaceae | unclassified |
| OTU_118 | 0 | 16.42 | 0.41 | 3.16 | | | 0.61 | | 30.82 | Bacteria | Bacteroidetes | Flavobacteriia | Flavobacteriales | Flavobacteriaceae | Winogradskyella |
| OTU_213 | 0 | 16.09 | 0.4 | 3.66 | | | 0.6 | | 31.42 | Bacteria | Bacteroidetes | [Saprospirae] | [Saprospirales] | Saprospiraceae | unclassified |
| OTU_112 | 3.16 | 18.86 | 0.39 | 2.13 | | | 0.59 | | 32.01 | Bacteria | Proteobacteria | Alphaproteobacteria | Rhodobacterales | Rhodobacteraceae | unclassified |
| OTU_189 | 0 | 15.37 | 0.38 | 2.93 | | | 0.57 | | 32.58 | Bacteria | Proteobacteria | Alphaproteobacteria | Kiloniellales | unclassified | unclassified |
| OTU_216 | 0 | 14.94 | 0.38 | 5.39 | | | 0.57 | | 33.15 | Bacteria | Bacteroidetes | Flavobacteriia | Flavobacteriales | Flavobacteriaceae | unclassified |
| OTU_80 | 14.5 | 0 | 0.38 | 1.32 | | | 0.56 | | 33.72 | Bacteria | Bacteroidetes | Flavobacteriia | Flavobacteriales | Flavobacteriaceae | unclassified |
| OTU_173 | 15.69 | 0.75 | 0.37 | 1.4 | | | 0.56 | | 34.27 | Bacteria | Bacteroidetes | Flavobacteriia | Flavobacteriales | Flavobacteriaceae | unclassified |
| OTU_42 | 17.43 | 5.51 | 0.36 | 1.5 | | | 0.53 | | 34.81 | Bacteria | Bacteroidetes | [Saprospirae] | [Saprospirales] | Saprospiraceae | unclassified |
| OTU_40 | 9.2 | 22.68 | 0.35 | 1.62 | | | 0.52 | | 35.32 | Bacteria | Proteobacteria | Alphaproteobacteria | Rhodobacterales | Rhodobacteraceae | unclassified |
| OTU_140 | 13.37 | 0 | 0.35 | 1.38 | | | 0.52 | | 35.84 | Bacteria | TM7 | unclassified | unclassified | unclassified | unclassified |
| OTU_401 | 1.8 | 15.24 | 0.34 | 3 | | | 0.51 | | 36.35 | Bacteria | Proteobacteria | Alphaproteobacteria | Rhodobacterales | Rhodobacteraceae | unclassified |
| OTU_291 | 13.06 | 0 | 0.34 | 1.44 | | | 0.51 | | 36.86 | Bacteria | Bacteroidetes | Flavobacteriia | Flavobacteriales | Flavobacteriaceae | Tenacibaculum |
| OTU_14 | 10.06 | 20.43 | 0.34 | 1.67 | | | 0.51 | | 37.37 | Bacteria | Proteobacteria | Alphaproteobacteria | Rhodobacterales | Rhodobacteraceae | unclassified |
| OTU_152 | 1.28 | 14.61 | 0.34 | 5.42 | | | 0.5 | | 37.87 | Bacteria | Bacteroidetes | Flavobacteriia | Flavobacteriales | Flavobacteriaceae | unclassified |
| OTU_94 | 0.33 | 13.56 | 0.33 | 3.18 | | | 0.5 | | 38.37 | Bacteria | Actinobacteria | Acidimicrobiia | Acidimicrobiales | wb1_P06 | unclassified |
| OTU_137 | 14.05 | 1.47 | 0.33 | 2.17 | | | 0.49 | | 38.86 | Bacteria | Proteobacteria | Gammaproteobacteria | Thiotrichales | Thiotrichaceae | Cocleimonas |
| OTU_210 | 0 | 12.68 | 0.33 | 2.05 | | | 0.49 | | 39.34 | Bacteria | Proteobacteria | Alphaproteobacteria | unclassified | unclassified | unclassified |
| OTU_3443 | 0 | 12.85 | 0.32 | 4.99 | | | 0.48 | | 39.83 | Bacteria | Proteobacteria | Alphaproteobacteria | unclassified | unclassified | unclassified |
| OTU_231 | 19.47 | 7.02 | 0.32 | 1.94 | | | 0.48 | | 40.31 | Bacteria | [Thermi] | Deinococci | Deinococcales | Trueperaceae | unclassified |
| OTU_71 | 1.38 | 14.24 | 0.32 | 1.19 | | | 0.48 | | 40.78 | Bacteria | Proteobacteria | Alphaproteobacteria | Rhodobacterales | Rhodobacteraceae | Phaeobacter |
| OTU_193 | 4.45 | 16.81 | 0.32 | 2.24 | | | 0.48 | | 41.26 | Bacteria | Proteobacteria | Alphaproteobacteria | Rhodobacterales | Rhodobacteraceae | unclassified |
| OTU_175 | 0.47 | 12.82 | 0.31 | 8.45 | | | 0.47 | | 41.73 | Bacteria | Bacteroidetes | [Saprospirae] | [Saprospirales] | Saprospiraceae | unclassified |
| OTU_82 | 2.58 | 14.93 | 0.31 | 1.98 | | | 0.47 | | 42.2 | Bacteria | Proteobacteria | Alphaproteobacteria | Rhodobacterales | Rhodobacteraceae | unclassified |
| OTU_211 | 0 | 12.07 | 0.31 | 3.27 | | | 0.46 | | 42.66 | Bacteria | Planctomycetes | Planctomycetia | Pirellulales | Pirellulaceae | unclassified |
| OTU_951 | 1.99 | 13.59 | 0.3 | 2.52 | | | 0.45 | | 43.11 | Bacteria | Proteobacteria | Alphaproteobacteria | Rhizobiales | Phyllobacteriaceae | unclassified |
| OTU_156 | 0 | 12.25 | 0.3 | 0.67 | | | 0.44 | | 43.55 | Bacteria | Proteobacteria | Alphaproteobacteria | Rhizobiales | Hyphomicrobiaceae | unclassified |
| OTU_163 | 0 | 11.25 | 0.29 | 1.23 | | | 0.44 | | 43.99 | Bacteria | Bacteroidetes | Flavobacteriia | Flavobacteriales | Flavobacteriaceae | unclassified |
| OTU_1 | 0.33 | 11.97 | 0.29 | 3.72 | | | 0.44 | | 44.42 | Bacteria | Proteobacteria | Alphaproteobacteria | Rhodobacterales | Rhodobacteraceae | unclassified |
| OTU_966 | 3.63 | 15.24 | 0.29 | 2.47 | | | 0.43 | | 44.86 | Bacteria | Proteobacteria | Alphaproteobacteria | Rhodobacterales | Rhodobacteraceae | Octadecabacter |
| OTU_103 | 10.72 | 20.9 | 0.29 | 1.52 | | | 0.43 | | 45.29 | Bacteria | Proteobacteria | Alphaproteobacteria | unclassified | unclassified | unclassified |
| OTU_296 | 0.33 | 11.5 | 0.28 | 8.2 | | | 0.42 | | 45.71 | Bacteria | Proteobacteria | Alphaproteobacteria | Rhodobacterales | Rhodobacteraceae | unclassified |
| OTU_72 | 15.97 | 4.94 | 0.28 | 1.66 | | | 0.42 | | 46.14 | Bacteria | Bacteroidetes | [Saprospirae] | [Saprospirales] | Saprospiraceae | unclassified |
| OTU_269 | 0.82 | 11.77 | 0.28 | 3.09 | | | 0.42 | | 46.56 | Bacteria | Bacteroidetes | Flavobacteriia | Flavobacteriales | Flavobacteriaceae | Actibacter |
| OTU_442 | 1.29 | 12.37 | 0.28 | 2.49 | | | 0.42 | | 46.97 | Bacteria | Proteobacteria | Gammaproteobacteria | Thiotrichales | Thiotrichaceae | Leucothrix |
| OTU_183 | 0 | 10.91 | 0.28 | 10.27 | | | 0.41 | | 47.38 | Bacteria | Bacteroidetes | Flavobacteriia | Flavobacteriales | Flavobacteriaceae | unclassified |
| OTU_171 | 10.82 | 0 | 0.27 | 2.57 | | | 0.41 | | 47.79 | Bacteria | Bacteroidetes | Flavobacteriia | Flavobacteriales | Flavobacteriaceae | Tenacibaculum |
| OTU_1991 | 0 | 10.89 | 0.27 | 1.48 | | | 0.41 | | 48.2 | Bacteria | Verrucomicrobia | Verrucomicrobiae | Verrucomicrobiales | Verrucomicrobiaceae | Haloferula |
| OTU_136 | 11.44 | 0.67 | 0.27 | 3.72 | | | 0.41 | | 48.61 | Bacteria | GN02 | BD1-5 | unclassified | unclassified | unclassified |
| OTU_3916 | 1.28 | 11.86 | 0.27 | 4.04 | | | 0.4 | | 49.01 | Bacteria | Proteobacteria | Alphaproteobacteria | Rhodobacterales | Rhodobacteraceae | unclassified |
| OTU_69 | 10.51 | 0 | 0.26 | 0.76 | | | 0.39 | | 49.4 | Bacteria | OD1 | ZB2 | unclassified | unclassified | unclassified |
| OTU_165 | 7.29 | 14.75 | 0.26 | 1.81 | | | 0.39 | | 49.79 | Bacteria | Bacteroidetes | Flavobacteriia | Flavobacteriales | Flavobacteriaceae | unclassified |
| OTU_275 | 0 | 10.32 | 0.26 | 3.09 | | | 0.39 | | 50.18 | Bacteria | Proteobacteria | unclassified | unclassified | unclassified | unclassified |
| OTU_369 | 10.38 | 0 | 0.26 | 4.5 | | | 0.39 | | 50.57 | Bacteria | Proteobacteria | Gammaproteobacteria | Thiotrichales | Thiotrichaceae | Leucothrix |
| OTU_204 | 6.75 | 16.78 | 0.26 | 1.8 | | | 0.39 | | 50.96 | Bacteria | Proteobacteria | Alphaproteobacteria | Rhodobacterales | Rhodobacteraceae | unclassified |
| OTU_3797 | 0.33 | 10.63 | 0.26 | 1.36 | | | 0.39 | | 51.34 | Bacteria | Proteobacteria | Alphaproteobacteria | Rhodobacterales | Rhodobacteraceae | unclassified |
| OTU_325 | 0 | 10.06 | 0.26 | 6.37 | | | 0.38 | | 51.73 | Bacteria | Bacteroidetes | [Saprospirae] | [Saprospirales] | Saprospiraceae | unclassified |
| OTU_23 | 11.91 | 18.74 | 0.26 | 1.84 | | | 0.38 | | 52.11 | Bacteria | Cyanobacteria | unclassified | unclassified | unclassified | unclassified |
| OTU_215 | 5.95 | 15.84 | 0.25 | 1.75 | | | 0.38 | | 52.49 | Bacteria | Proteobacteria | Alphaproteobacteria | Rhodobacterales | Rhodobacteraceae | unclassified |
| OTU_132 | 11.96 | 14.89 | 0.25 | 1.29 | | | 0.38 | | 52.86 | Bacteria | Proteobacteria | Alphaproteobacteria | Rhodobacterales | Hyphomonadaceae | unclassified |
| OTU_247 | 9.78 | 0 | 0.25 | 1.03 | | | 0.37 | | 53.24 | Bacteria | SR1 | unclassified | unclassified | unclassified | unclassified |
| OTU_108 | 0.82 | 10.66 | 0.25 | 2.67 | | | 0.37 | | 53.61 | Bacteria | Proteobacteria | Alphaproteobacteria | Kiloniellales | unclassified | unclassified |
| OTU_129 | 12.74 | 4.6 | 0.25 | 1.63 | | | 0.37 | | 53.98 | Bacteria | Proteobacteria | Alphaproteobacteria | Rhodobacterales | Rhodobacteraceae | unclassified |
| OTU_180 | 0 | 9.2 | 0.25 | 0.74 | | | 0.37 | | 54.34 | Bacteria | Bacteroidetes | unclassified | unclassified | unclassified | unclassified |
| OTU_214 | 1.28 | 11.14 | 0.24 | 1.72 | | | 0.36 | | 54.71 | Bacteria | Proteobacteria | Alphaproteobacteria | Rhodobacterales | Rhodobacteraceae | unclassified |
| OTU_131 | 9.62 | 0 | 0.24 | 0.7 | | | 0.36 | | 55.07 | Bacteria | Bacteroidetes | [Saprospirae] | [Saprospirales] | Saprospiraceae | unclassified |
| OTU_264 | 0 | 9.6 | 0.24 | 1.39 | | | 0.36 | | 55.43 | Bacteria | Bacteroidetes | Flavobacteriia | Flavobacteriales | Flavobacteriaceae | Tenacibaculum |
| OTU_16 | 18.69 | 12.31 | 0.24 | 1.26 | | | 0.36 | | 55.79 | Bacteria | Actinobacteria | Acidimicrobiia | Acidimicrobiales | unclassified | unclassified |
| OTU_66 | 4.25 | 12.67 | 0.24 | 1.05 | | | 0.36 | | 56.15 | Bacteria | Bacteroidetes | Flavobacteriia | Flavobacteriales | Flavobacteriaceae | unclassified |
| OTU_245 | 0 | 9.35 | 0.24 | 3.4 | | | 0.36 | | 56.51 | Bacteria | Proteobacteria | Alphaproteobacteria | Rhodobacterales | Rhodobacteraceae | Roseovarius |
| OTU_337 | 3.15 | 12.34 | 0.24 | 1.73 | | | 0.35 | | 56.86 | Bacteria | Proteobacteria | Alphaproteobacteria | Rhodobacterales | Rhodobacteraceae | unclassified |
| OTU_447 | 0 | 9.18 | 0.24 | 1.64 | | | 0.35 | | 57.22 | Bacteria | Proteobacteria | Alphaproteobacteria | Rhodospirillales | unclassified | unclassified |
| OTU_328 | 0 | 9.26 | 0.24 | 5.18 | | | 0.35 | | 57.57 | Bacteria | Proteobacteria | Alphaproteobacteria | unclassified | unclassified | unclassified |
| OTU_3 | 1.33 | 10.58 | 0.23 | 4.12 | | | 0.35 | | 57.92 | Bacteria | Proteobacteria | Alphaproteobacteria | Rhodobacterales | Rhodobacteraceae | Oceanibulbus |
| OTU_90 | 0 | 9.3 | 0.23 | 4.12 | | | 0.35 | | 58.27 | Bacteria | Verrucomicrobia | Verrucomicrobiae | Verrucomicrobiales | Verrucomicrobiaceae | unclassified |
| OTU_2296 | 9.15 | 0 | 0.23 | 3.97 | | | 0.35 | | 58.61 | Bacteria | Bacteroidetes | [Saprospirae] | [Saprospirales] | Saprospiraceae | unclassified |
| OTU_153 | 5.16 | 14.27 | 0.23 | 1.53 | | | 0.35 | | 58.96 | Bacteria | Bacteroidetes | Flavobacteriia | Flavobacteriales | Flavobacteriaceae | unclassified |
| OTU_143 | 8.75 | 0 | 0.23 | 1.03 | | | 0.35 | | 59.31 | Bacteria | Bacteroidetes | [Saprospirae] | [Saprospirales] | Saprospiraceae | unclassified |
| OTU_147 | 0 | 9.32 | 0.23 | 1.03 | | | 0.34 | | 59.65 | Bacteria | Bacteroidetes | Flavobacteriia | Flavobacteriales | Flavobacteriaceae | unclassified |
| OTU_3793 | 0.33 | 9.03 | 0.22 | 4.13 | | | 0.33 | | 59.98 | Bacteria | Proteobacteria | Alphaproteobacteria | Rhodobacterales | Rhodobacteraceae | unclassified |
| OTU_149 | 1 | 9.83 | 0.22 | 1.55 | | | 0.33 | | 60.31 | Bacteria | Verrucomicrobia | Verrucomicrobiae | Verrucomicrobiales | Verrucomicrobiaceae | unclassified |

| **(B) SIMPER analysis bacteria in the surrounding seawater** | | | | | | | | | | | |  |
| --- | --- | --- | --- | --- | --- | --- | --- | --- | --- | --- | --- | --- |
| Groups t1 & t6 | |  |  |  |  |  |  |  |  |  |  |  |
| Average dissimilarity = 32.59 | | |  |  |  |  |  |  |  |  |  |  |
|  |  |  |  |  |  |  |  |  |  |  |  |  |
|  | Group t1 | Group t6 |  |  |  |  |  |  |  |  |  |  |
| OTU | Av.Abund | Av.Abund | Av.Diss | Diss/SD | Contrib% | Cum.% | Kingdom | Phylum | Class | Order | Family | Genus |
| OTU_105 | 44.2 | 3.74 | 1.56 | 10.4 | 4.8 | 4.8 | Bacteria | Proteobacteria | Alphaproteobacteria | unclassified | unclassified | unclassified |
| OTU_43 | 37.83 | 0 | 1.44 | 2.81 | 4.42 | 9.23 | Bacteria | Proteobacteria | Alphaproteobacteria | Rhodobacterales | Rhodobacteraceae | unclassified |
| OTU_63 | 34.99 | 2 | 1.27 | 4.42 | 3.89 | 13.11 | Bacteria | Proteobacteria | Alphaproteobacteria | Rhodobacterales | Rhodobacteraceae | Octadecabacter |
| OTU_5 | 24.41 | 55.67 | 1.24 | 2.69 | 3.81 | 16.92 | Bacteria | Proteobacteria | Alphaproteobacteria | Rickettsiales | Pelagibacteraceae | unclassified |
| OTU_1 | 44.77 | 69.43 | 0.97 | 3.97 | 2.98 | 19.9 | Bacteria | Proteobacteria | Alphaproteobacteria | Rhodobacterales | Rhodobacteraceae | unclassified |
| OTU_1155 | 59.13 | 34.03 | 0.97 | 13.49 | 2.98 | 22.88 | Bacteria | Proteobacteria | Alphaproteobacteria | Rhodobacterales | Rhodobacteraceae | unclassified |
| OTU_20 | 45.88 | 21.38 | 0.95 | 9.29 | 2.91 | 25.79 | Bacteria | Proteobacteria | Alphaproteobacteria | unclassified | unclassified | unclassified |
| OTU_3916 | 29.9 | 5.92 | 0.92 | 5.48 | 2.83 | 28.62 | Bacteria | Proteobacteria | Alphaproteobacteria | Rhodobacterales | Rhodobacteraceae | unclassified |
| OTU_58 | 38.01 | 14.76 | 0.9 | 5.99 | 2.75 | 31.37 | Bacteria | Bacteroidetes | Flavobacteriia | Flavobacteriales | Flavobacteriaceae | Flavobacterium |
| OTU_139 | 26.65 | 4.8 | 0.84 | 4.65 | 2.58 | 33.94 | Bacteria | Proteobacteria | Betaproteobacteria | Methylophilales | Methylophilaceae | unclassified |
| OTU_78 | 1 | 19.67 | 0.73 | 5.4 | 2.25 | 36.19 | Bacteria | Proteobacteria | Gammaproteobacteria | Oceanospirillales | Halomounclassifieddaceae | Candidatus Portiera |
| OTU_220 | 4.44 | 22.49 | 0.69 | 4.12 | 2.12 | 38.32 | Bacteria | Proteobacteria | Alphaproteobacteria | Rickettsiales | AEGEAN_112 | unclassified |
| OTU_50 | 6.2 | 22.49 | 0.65 | 2.43 | 1.99 | 40.31 | Bacteria | Bacteroidetes | [Rhodothermi] | [Rhodothermales] | [Balneolaceae] | Balneola |
| OTU_2 | 141.11 | 125.03 | 0.59 | 1.08 | 1.83 | 42.13 | Bacteria | Proteobacteria | Alphaproteobacteria | Rickettsiales | Pelagibacteraceae | unclassified |
| OTU_188 | 5.96 | 20.27 | 0.58 | 1.48 | 1.77 | 43.9 | Bacteria | Proteobacteria | Alphaproteobacteria | Rickettsiales | Pelagibacteraceae | unclassified |
| OTU_707 | 1.22 | 15.65 | 0.56 | 102.06 | 1.72 | 45.62 | Bacteria | Proteobacteria | Alphaproteobacteria | Rickettsiales | Pelagibacteraceae | unclassified |
| OTU_75 | 3.05 | 16.06 | 0.52 | 3.04 | 1.58 | 47.21 | Bacteria | Bacteroidetes | [Rhodothermi] | [Rhodothermales] | [Balneolaceae] | KSA1 |
| OTU_1037 | 0 | 12.96 | 0.51 | 9.06 | 1.56 | 48.76 | Bacteria | Proteobacteria | Deltaproteobacteria | Sva0853 | unclassified | unclassified |
| OTU_3 | 24.29 | 35.74 | 0.47 | 1.07 | 1.44 | 50.2 | Bacteria | Proteobacteria | Alphaproteobacteria | Rhodobacterales | Rhodobacteraceae | Oceanibulbus |
| OTU_107 | 17.04 | 5.1 | 0.46 | 5.48 | 1.41 | 51.61 | Bacteria | Bacteroidetes | Flavobacteriia | Flavobacteriales | Flavobacteriaceae | unclassified |
| OTU_99 | 9 | 20.15 | 0.44 | 4.89 | 1.35 | 52.96 | Bacteria | Bacteroidetes | Flavobacteriia | Flavobacteriales | Flavobacteriaceae | unclassified |
| OTU_604 | 0 | 11.14 | 0.44 | 9.06 | 1.34 | 54.29 | Bacteria | Proteobacteria | Alphaproteobacteria | Rhodobacterales | Rhodobacteraceae | unclassified |
| OTU_232 | 16.91 | 5.66 | 0.43 | 6.19 | 1.33 | 55.62 | Bacteria | Proteobacteria | Betaproteobacteria | Methylophilales | Methylophilaceae | unclassified |
| OTU_542 | 0 | 10.95 | 0.43 | 9.06 | 1.31 | 56.94 | Bacteria | SBR1093 | A712011 | unclassified | unclassified | unclassified |
| OTU_698 | 0 | 10.58 | 0.41 | 9.06 | 1.27 | 58.21 | Bacteria | Proteobacteria | Alphaproteobacteria | Rickettsiales | unclassified | unclassified |
| OTU_258 | 13.92 | 3.16 | 0.4 | 1.2 | 1.23 | 59.43 | Bacteria | Proteobacteria | Gammaproteobacteria | unclassified | unclassified | unclassified |
| OTU_356 | 11.17 | 1 | 0.4 | 5.43 | 1.23 | 60.66 | Bacteria | Proteobacteria | Gammaproteobacteria | unclassified | unclassified | unclassified |
